# Agrimoniin ameliorates intrapulmonary angiogenesis and improves hypoxemia in hepatopulmonary syndrome via PGC-1α activation and glycolysis down-regulation

**DOI:** 10.1016/j.gendis.2025.101941

**Published:** 2025-11-18

**Authors:** Ziyang Zeng, Zhiyong Yang, Yuhao Lei, Meiyu Zhou, Lin Chen, Yang Chen, Xianfeng Wu, Huiling Cao, Chunyong Yang, Xiaobo Wang, Karine Belguise, Yujie Li, Bin Yi

**Affiliations:** a Department of Anesthesiology, The First Affiliated Hospital (Southwest Hospital) of Army Medical University, Key Laboratory of Perioperative Multi-organ Protection and Intelligent Anesthesia of Chongqing Municipal Health Commission, Chongqing 400038, China; b Department of Anesthesiology, Chongqing Traditional Chinese Medicine Hospital, Chongqing 400021, China; c Department of Cardiovascular Medicine, Center for Circadian Metabolism and Cardiovascular Disease, The First Affiliated Hospital (Southwest Hospital) of Army Medical University, Ministry of Education Key Laboratory of Geriatric Cardiovascular and Cerebrovascular Disease, Chongqing 400038, China; d Department of Anesthesia, The Seventh People's Hospital of Chongqing (The Affiliated Central Hospital of Chongqing University of Technology), Chongqing 400054, China; e Molecular, Cellular and Developmental Biology Department (MCD), Centre de Biologie Intégrative (CBI), Université de Toulouse, CNRS, UPS, Toulouse 31062, France

**Keywords:** Agrimoniin, Angiogenesis, Glycolysis, Hepatopulmonary syndrome, Mitochondrialdysfunction, PGC-1α

## Abstract

Hepatopulmonary syndrome (HPS) is a condition characterized by pulmonary angiogenesis and refractory hypoxemia, often seen in patients with chronic liver disease. Its unclear mechanism means that liver transplantation is the only effective therapy. Agrimoniin, a compound from *Pilosa ledeb*, shows potential in protecting against liver cirrhosis via anti-angiogenic and anti-glycolytic effects. This study investigates agrimoniin as a potential integrated therapy for HPS-related liver and lung dysfunction. Using transcriptome data and an ICU cohort, we analyzed the role of glycolysis in chronic liver disease progression. HPS rats were established via common bile duct ligation, and serum metabolites were measured. The oxygen consumption rate and extracellular acidification rate were also detected. Rats were treated with agrimoniin (3 mg/kg/day or 8 mg/kg/day) at the early stage of HPS. Our results showed that imbalanced oxidative phosphorylation and glycolysis correlated with chronic liver disease progression and poorer outcomes. Decreased oxygen consumption rate and increased extracellular acidification rate, as well as increased glycolysis, were observed in the HPS group. Agrimoniin treatment improved liver and lung function by inhibiting pathological angiogenesis and glycolysis. Through TCM suite analysis, molecular docking, and dynamics simulations, PGC-1α was identified as a potential target of agrimoniin. Inhibiting PGC-1α blocked agrimoniin's benefits on angiogenesis and glycolysis flux. Thus, agrimoniin may be a potential integrated therapy for HPS by activating PGC-1α to inhibit glycolysis and angiogenesis.

## Introduction

Hepatopulmonary syndrome (HPS) is characterized by intrapulmonary vascular dilatations and refractory hypoxemia that occurs in the context of chronic liver disease (CLD), with an incidence rate of approximately 5%–30%.^1^ Previously, lots of treatments have been tried; however, till now, the only treatment remains liver transplantation.^2^ It may be due to existing therapies focusing only on lung pathology without integrating the treatment of both the liver and the lung. The mechanisms underlying HPS are highly complex.^3^ Pulmonary microvascular dilation and hepatopulmonary angiogenesis have been established as the key pathogenic features of HPS.^4^ While the liver's central role in glucose homeostasis is well-documented, the impact of disrupted glucose homeostasis on the progression of liver disease and its associated complications has only recently been recognized.^5^^,^^6^ Moreover, glycolysis induced by mitochondrial dysfunction was also reported to play an important role in the progression of CLD.^7^^,^^8^ Specifically, an elevation in glycolytic flux has been noted to augment hepatic angiogenesis.^9^ Furthermore, glycolysis has been implicated in the pathophysiology of diverse diseases such as angiogenic disorders.^10^ Therefore, modulation of glucose metabolism to mitigate pulmonary angiogenesis may constitute a novel therapeutic strategy for HPS. Agrimoniin, a polyphenolic compound derived from *Pilosa ledeb*, undergoes metabolism by human intestinal flora and exhibits a broad spectrum of health benefits, encompassing anti-inflammatory, anti-angiogenesis, and anti-glycolysis.^11^ Literature reports indicate that agrimoniin is involved in glucose metabolism. Specifically, agrimoniin rectifies abnormal glucose metabolism and improves glucose homeostasis by mitigating insulin resistance in mice.^12^ More importantly, agrimoniin sustains hepatocyte mitochondrial functions under a spectrum of pathological conditions.^13^ However, most studies have focused on tumor contexts^14^^,^^15^ or pure liver protection.^16^^,^^17^ Few studies have investigated the effects of agrimoniin on extrahepatic organ injury induced by liver disease, such as HPS. Given that agrimoniin is a natural product with a history of traditional use, it offers a relatively safe and promising therapeutic option for HPS. Collectively, based on the hepatoprotective effects of agrimoniin via regulation of glycolysis and angiogenesis, the present study aims to evaluate the effects and potential underlying mechanisms of agrimoniin as an integrated therapy for liver and lung dysfunction in HPS.

## Materials and methods

### Study cohorts and data collection

To investigate the association between glycolysis and clinical outcome in patients with CLD, we extracted data from the multi-parameter intelligent monitoring in intensive care (MIMIC IV) database (version 2.2).^18^ The requirement for individual patient consent was waived by the Institutional Review Board at the BIDMC. All the data were accessed via a data use agreement between PhysioNet, a National Institutes of Health–supported data repository (https://www.physionet.org/, Certification Number: 28,341,490). The inclusion criteria were as follows: i) age older than 18 years old; ii) patients with chronic liver disease, including but not limited to cirrhosis, acute on chronic liver failure, liver cancer, and chronic hepatitis. The following indicators are considered: Charlson comorbidity index (CCI), white blood cell count, PaO_2_, oxygen saturation (SO_2_), oxygenation index, platelet count, alanine aminotransferase, aspartate aminotransferase, alkaline phosphatase, glucose, hemoglobin, international normalized ratio, serum lactate (Lac), lactate dehydrogenase (LDH), prothrombin time, activated partial thromboplastin time, admission time, discharge time, time of death, and ICU duration. Data with glucose levels exceeding 33.3 mmol/L (*i.e.*, 599.4 mg/dL) and PaO_2_ levels greater than 120 mmHg were excluded. After data quality control, the maximum, minimum, and average values of the above laboratory indicators during hospitalization were calculated. For all these patients, the outcomes were defined as in-hospital mortality and respiratory failure during hospitalization. Respiratory failure during hospitalization is defined as the occurrence of PaO_2_ < 60 mmHg or PaCO_2_ > 50 mmHg for any reason during hospital stay. The statistical analysis between the two groups was conducted using the compareGroups package.^19^

To identify key pathways and targets to angiogenesis, glycometabolism, and mitochondria in decompensated cirrhosis via data mining from transcriptome datasets. We extracted data from the Gene Expression Omnibus (GEO, http://www.ncbi.nlm.nih.gov/geo/). Herein, we analyzed GSE139602^9^ to investigate changes in glycometabolism-related pathways and gene expression during the progression of compensatory/decompensated/chronic acute liver failure patients, and analyzed GSE208637^20^ to verify differential interest gene expression. Differential pathway and gene analyses were performed using the limma package,^21^ with significant pathways and genes identified based on adjusted *p*-values. Fuzzy clustering^22^ was applied to analyze pathway trends. Potential targets of agrimoniin were predicted by TCM suite (http://tcm-suite.aimicrobiome.cn/).^23^ The affinity between agrimoniin and potential targets was calculated by the CSatDTA online tool.^24^ Then CB-DOCK,^25^ a pipeline model that can automatically remove water molecules and heteroatoms from the input structure, was applied to perform molecular docking prediction analysis between agrimoniin and potential targets. To further evaluate the complex by CB-DOCK, molecular dynamics simulations were performed using GROMACS software with the AMBER 99SB-ILDN force field for proteins, GAFF for agrimoniin, and TIP3P for water. The system was solvated, energy-minimized, equilibrated, and simulated for 100 ns.

### Animals

All experimental procedures were approved by the Committee on Animal Research of Chongqing Traditional Chinese Medicine Hospital (2021-DWSY-ZZY). All experiments were conducted in accordance with the principles of laboratory animal care provided by the National Institutes of Health.

Sprague–Dawley (SD) and specific-pathogen-free male rats, weighing 230–330 g, were purchased from the Laboratory Animal Center of the Third Military University. Common bile duct ligation (CBDL) in the rat has served as a common experimental HPS model as described.^26^ Agrimoniin (C82H54O52, HPLC > 98%, CAS#491-67-8, Yuanye Bio-Technology, China) was purchased from Yuanye Bio-Technology. As shown in Figure 3A, all rats were randomly assigned to four groups as follows: i) control group (control; *n* = 20); ii) CBDL groups; (CBDL, *n* = 20); iii) CBDL rats with agrimoniin at low dosage (A–L; *n* = 20); iv) CBDL rats with agrimoniin at high dosage (A–H; *n* = 20). 2 weeks after CBDL, agrimoniin was administered intraperitoneally for 3 weeks (3 mg/kg/day and 8 mg/kg/day, respectively). The tissues were collected and weighed 3 weeks after intervention. The saline solution was used as a placebo. Rats were anesthetized with isoflurane.

**Figure 1:**
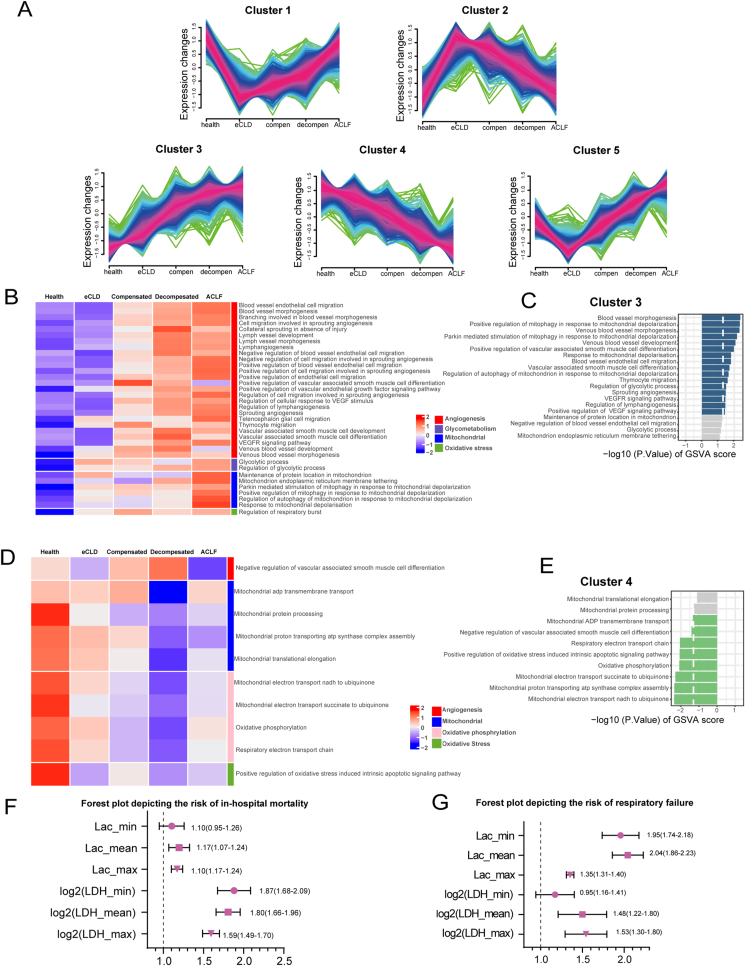
OXPHOS/glycolysis imbalance drives chronic liver disease progression. **(A)** The Series Test of Cluster results in bioinformatics: the trends showed no redundancy and were rational. Cluster 1 shows a down-regulation in liver disease patients compared with healthy volunteers, followed by an up-regulation with increasing disease severity. Cluster 2 exhibits a down-regulation in early-stage liver disease, which persists and intensifies with severity. Cluster 3 displays an up-regulation correlated with severity, while cluster 4 demonstrates a down-regulation. Cluster 5 initially shows a down-regulation in early-stage liver disease, followed by an up-regulation with severity. **(B)** Representation of Heat maps of the interested pathways for GSVA cluster 3. **(C)** The top variations between ACLF and healthy volunteers with the most significant disparities in cluster 3. **(D)** Representation of Heat maps of the interested pathways for GSVA cluster 4. **(E)** The top variations between ACLF and healthy volunteers with the most significant disparities in cluster 4. **(F)** Lac and LDH are risk stratification models for predicting in-hospital mortality in patients with liver disease. **(G)** Lac and LDH are biomarkers used to evaluate respiratory risk in patients with liver disease. R Statistical Software and the package ComplexHeatmap were used.

**Figure 2:**
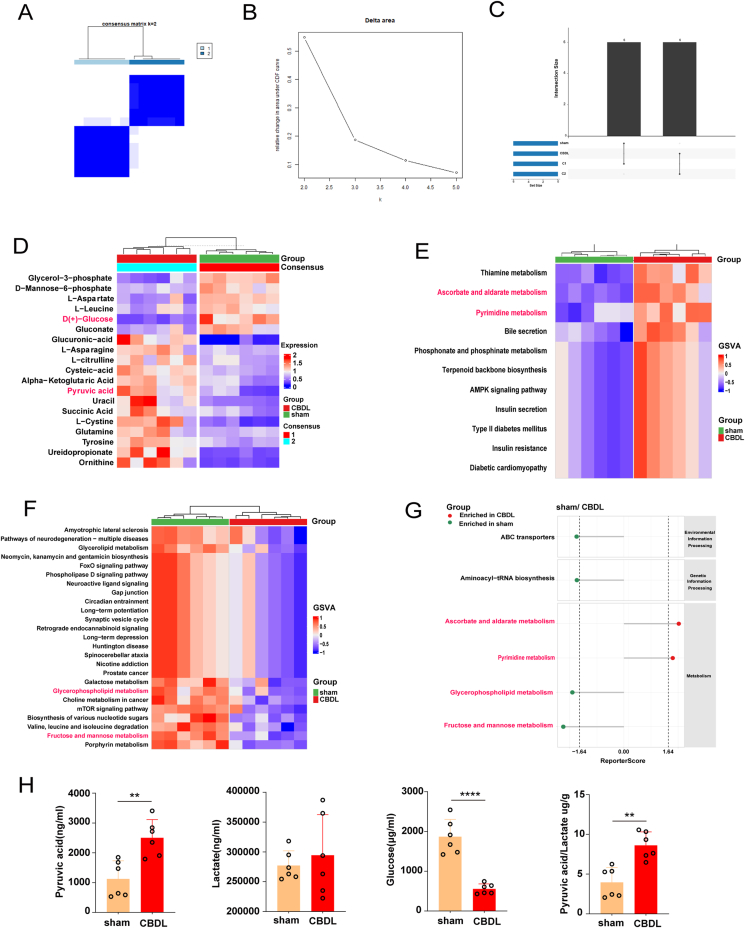
The serum energy metabolites analysis. **(A)** The unsupervised consensus clustering of the serum energy metabolites, when *k* = 2. **(B)** The changes of the delta area along with k. **(C)** The UpSet Diagrams of the clusters and the original group. **(D)** The heatmap of important serum metabolites calculated by clustering analysis. **(E, F)** The significant up (E) and down (F) pathways between the CBDL and sham group by GSVA analysis. **(G)** The enriched pathways calculated by GRSA analysis. **(H)** The concentration of important serum metabolites in the sham and CBDL group. ∗*p* < 0.05, ∗∗*p* < 0.01, and ∗∗∗∗*p* < 0.0001.

**Figure 3:**
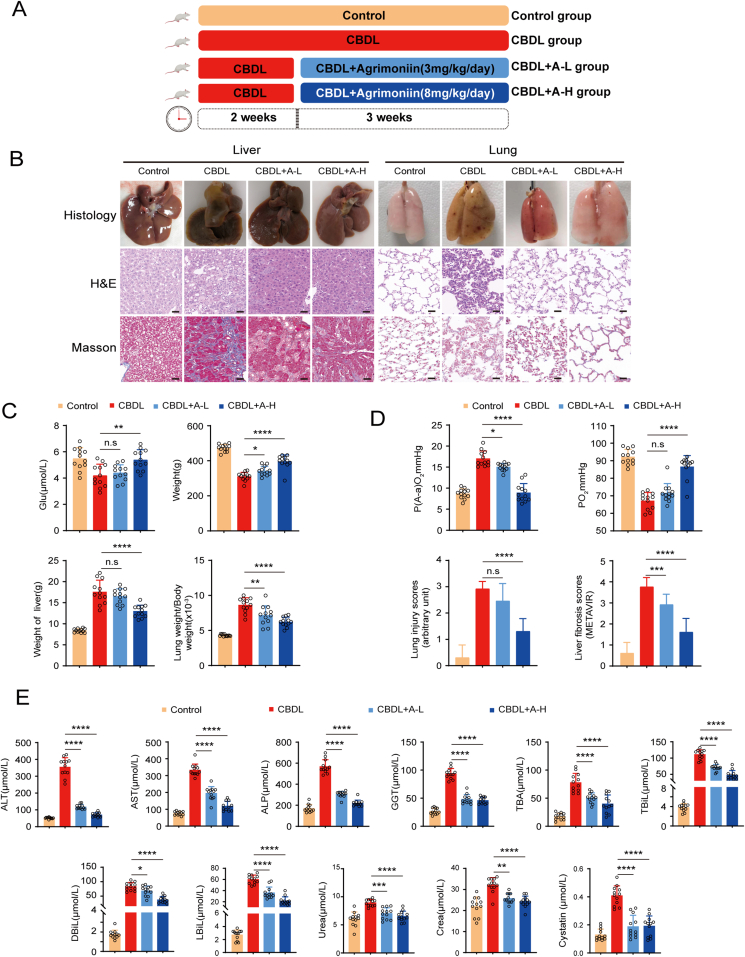
The protective effect of agrimoniin on the CBDL rats. **(A)** The detailed diagram of the experimental process. **(B)** Representative images of histology, HE, and Masson staining of liver and lung tissue from each group (*n* = 12 per group). **(C)** Effects of agrimoniin on blood glucose, body weight, and liver and lung organ weight in different groups (*n* = 12 per group). **(D)** Arterial blood gas analysis results, lung injury score, and liver fibrosis METAVIR scores of rats in different intervention groups (*n* = 12 per group). **(E)** Liver enzyme and bilirubin metabolism in different intervention groups (*n* = 12 per group). Scale bar, 50 μm ∗*p* < 0.05, ∗∗*p* < 0.01, ∗∗∗*p* < 0.001, and ∗∗∗∗*p* < 0.0001.

### Detection of targeted metabolites

The serum samples from the sham and CBDL groups were tested (*n* = 6 for each group). The tested compounds were listed in Table S1. The sample was vortexed for 10 s, and 50 μL was mixed with 250 μL of 20% acetonitrile/methanol, vortexed for 3 min, and centrifuged at 12,000 r/min at 4 °C for 10 min. The supernatant was stored at −20 °C for 30 min and then centrifuged again under the same conditions. Finally, 180 μL of the supernatant was transferred through a protein precipitation plate for liquid chromatography-mass spectrometry (LC-MS) analysis. The sample extracts were analyzed using a Waters ACQUITY H-Class UPLC coupled with a QTRAP 6500 plus LC-MS/MS system. The UPLC conditions included an ACQUITY UPLC BEH Amide column (2.1 × 100 mm, 1.7 μm) with a gradient of 95% B (0–1.2 min), 70% B (8 min), 50% B (9–11 min), and back to 95% B (11.1–15 min) at a flow rate of 0.4 mL/min and 40 °C. The electrospray ionization-mass spectrometry (ESI-MS)/MS parameters were set to source temperature 550 °C, ion spray voltage 5500 V (positive) and −4500 V (negative), and curtain gas 35 psi, with metabolites analyzed by scheduled MRM using Analyst 1.6.3 and Multiquant 3.0.3 software. The detailed information was described in the supplementary materials.

### Metabolism analysis

After the data were scaled to unit variance, unsupervised clustering analysis was performed by the ConsensusClusterPlus package.^27^ The important metabolites were determined by the mean levels of metabolites and the significance (*p*-value) across different clustering results. The differential pathway analysis was conducted by Gene Set Variation Analysis (GSVA) via the GSVA package.^28^ The enrichment of pathways was accomplished by Generalized Reporter Score-based Analysis (GRSA) via the ReporterScore package.^29^ We also used UpSetR^30^ and ComplexHeatmap^31^ for visualization.

### Biochemistry and blood gas analysis

Serum samples were harvested and centrifuged at 4000 r/min at 4 °C for 10 min. The resultant serum was analyzed using an automatic biochemistry analyzer (AU5400, Olympus, Japan). In addition, arterial blood samples were collected from the aorta ventralis and subsequently analyzed for arterial blood gas using an ABL 700 radiometer (Radiometer Copenhagen, Denmark).

### Histology and morphometry

Liver and lung tissues were fixed with 10% formalin for 24 h and embedded in paraffin. The 4 μm sections were cut from paraffin-embedded liver and lung tissues. Sections were stained with periodic acid-Schiff and Masson-trichrome staining for histology analysis.

### Vascular casting of lungs for micro-CT imaging and reconstruction

The vascular casting of lungs followed the work by Kozel et al.^32^ On the harvest day, the rat was arranged in a supine position and secured all four limbs to the board with tape. After exposing lungs and trachea, pulmonary artery catheterization, and blood perfusion, the microfil (MV-122) contrast agents were injected until the lobes were filled completely down to the capillary level. The lung was then dissected and placed in formalin overnight. The lung was placed on the scanning bed of micro-CT (SkyScan1272, Bruker) for scanning. The parameters were as follows: source voltage (kV) = 65; source current (μA) = 153; image pixel size (μm) = 20.022575. The image reconstruction was conducted by NRecon (Version 1.7.4.6), and the visualization of vascular reconstruction was conducted by CT vox (version 3.3.1).

### Tissue protein extraction

Samples of 30 mg were placed on the purification column and twisted 50–60 times with a plastic grinding rod. Then, 400 μL of denaturing lysate, 4 μL of protease inhibitors, and 4 μL of phosphatase inhibitors were added. The sample was then further ground 60 times. The purification column was covered and incubated at room temperature for 10 min, followed by centrifugation at 15,000 rpm for 2 min. The concentration of total protein was quantified using a protein assay kit (Thermos, Pierce™ BCA Protein Assay Kits, 23,225). Equivalent protein concentration in each sample was prepared and loaded for Western blotting analysis.

### Cell culture and transfection with PGC-1α small interfering RNA

Pulmonary microvascular endothelial cells (PMVECs) were sourced from the BeNa Culture Collection (Beijing, China) and cultured in Dulbecco's modified Eagle medium (DMEM, Hyclone, USA) containing 10% fetal bovine serum (Moregate, Bulimba, Australia) and 1% penicillin-streptomycin (Beyotime, Shanghai, China) under standard conditions. Peroxisome proliferator-activated receptor-γ coactivator-1α (PGC-1α)-specific small interfering RNA (siRNA; 5′-CAACUUCAGUAAUGAACAA-3) was synthesized and transfected into PMVECs using a transfection reagent according to the manufacturer's protocol. To establish the CBDL model *in vitro*, cells were incubated in DMEM containing 5% CBDL rat serum for 48 h. PMVECs incubated with 5% normal rat serum were used as a control.

### Scratch migration assay

PMVECs were digested and seeded into the 6-well plates (5 × 10^5^ cells/well). When the cells reached 90% confluence, a vertical scratch was made using a 20 μL sterile plastic pipette tip in the middle of each well. After 24 h incubation, photographs were captured at 0 h and 24 h after the scratch assay. The width of the scratch was measured using a light microscope (IX71, OLYMPUS, Japan).

### Tube formation assay

PMVECs were cultured in serum-free endothelial basal medium (EBM)-2 for 24 h, and Matrigel (Corning, USA) was thawed at 4 °C overnight. The 48-well plate was coated with Matrigel (180 μL per well) and incubated for 30 min. PMVECs (2 × 10^5^ cells/well) were seeded on the layer of polymerized Matrigel. After 4 h incubation, the tube formation of PMVECs was observed using a light microscope (IX71, OLYMPUS, Japan).

### Immunohistochemical and immunofluorescence staining

For DAB staining *in vivo*, 4-μm-thick paraffin sections were first dewaxed and subjected to heat-mediated antigen retrieval. Sections were exposed to 3% H_2_O_2_ in methanol for 30 min to quench endogenous peroxidases and then incubated with serum, followed by incubation with the primary antibodies at 4 °C overnight. After washing with phosphate-buffered saline (PBS), the slides were incubated with secondary (horseradish peroxidase-labeled goat anti-rabbit IgG (H + L) antibodies. The antibody staining patterns were visualized with DAB staining. The nucleus was counterstained with hematoxylin and then sealed with neutral resin. Ten representative regions per section were randomly selected by an assessor blinded to the treatment groups.

For fluorescence staining *in vivo*, 4-μm-thick sections were incubated with primary antibody at 4 °C overnight. After washing with PBS, the slides were incubated with fluorochrome-conjugated secondary horseradish peroxidase-labeled goat anti-rabbit IgG (H + L) antibodies. The antibody staining patterns were visualized with fluorescence. Sections were counterstained with Cy3-labeled goat anti-rabbit IgG (H + L) and FITC-labeled goat anti-rabbit IgG (H + L) antibodies. DAPI (4,6-diamidino-2-phenylindole, fluorescence staining) stains the nucleus. Sections were examined using the Axio Imager A2 (ZEISS, Germany) microscope. Immunofluorescence was quantified using ImageJ (National Institutes of Health). For each immunofluorescence slice, five randomly selected fields were chosen by Pannoramic MIDI (3DHISTECH, Hungary). The number of CD31-positive cells per high-power field was counted by Image-Pro Plus software (version 6.0, Media Cybernetics Inc, USA). Table S2 provides more information on antibodies.

### ELISA

The concentrations of vascular endothelial growth factor (VEGF; Shanghai Jonlnbio, catalog: JL21369), placental growth factor (PLGF; Shanghai Jonlnbio, catalog: JL11559), 6-phosphofructo-2-kinase/fructose-2,6-biphosphatase 3 (PFKFB3) (FineTest, catalog: ER2066), PGC-1α (FineTest, catalog: ER0455), LDH (Shanghai Jonlnbio, catalog: JL-T1070), pyruvate (Shanghai Jonlnbio, catalog: JL-T0763), and lactic acid (Shanghai Jonlnbio, catalog: JL-T1068) were examined with enzyme-linked immunosorbent assay (ELISA) kits according to the manufacturer's instructions.

### Laser scanning confocal microscopy

For immunofluorescence staining, 4-μm-thick sections were incubated with anti-PFKFB3 (1:100, ab181861, Abcam) at 4 °C overnight. Sections were counterstained with Cy3-labeled goat anti-rabbit IgG (H + L) antibody. After washing with PBS, the slides were incubated with anti-PGC-1α (1:100, ab191838, Abcam) at 4 °C overnight and incubated with FITC-labeled goat anti-rabbit IgG (H + L) antibody. DAPI (4,6-diamidino-2-phenylindole, fluorescence staining) stains the nucleus. Sections were examined using a Zeiss laser scanning confocal (ZEISS LSM880, Germany). For each immunofluorescence slice, five randomly selected fields were chosen by Pannoramic MIDI (3DHISTECH, Hungary). The number of PGC-1α-positive cells per field was counted by Image-Pro Plus software (version 6.0, Media Cybernetics Inc, USA).

### Quantification of mitochondrial DNA levels and DNA damage

Mitochondrial DNA (mtDNA) levels were determined using quantitative PCR. Therefore, we determined the cycle threshold (CT) of NADH dehydrogenase 1 (ND1) and nuclear gene beta-2 microglobulin (β2M). Oligonucleotide primers (Sangon Biotech, China) were designed using Clone Manager 9 software and were validated by assessing efficiency, melting, and temperature curves using the CFX384 Real-Time system (Bio-Rad, California, USA). Amplification of the DNA was performed using the following thermal profile: 95 °C for 2 min, followed by 40 cycles of 95 °C for 15 s and 61 °C for 60 s. All reactions were carried out in duplicate, and the obtained threshold cycles (CT) values were averaged. A standard curve was used to determine the efficiency, linear range, and reproducibility of the quantitative PCR assay. The difference in the CT value between ND1 and β2M was used to quantify the relative abundance of the mitochondrial genome.

mtDNA damage was assessed by PCR, by which we measured mitochondrial damage by performing a long-range PCR, whereby the long fragment of 10 kb is relatively more prone to damage as compared with the short fragment of 200 bp. Consequently, mtDNA damage can be measured by a relative decrease in the amount of the long fragment as compared with the short mtDNA fragment. The ratio of the intensity of the short stable fragment to the long unstable fragment was calculated to quantify mtDNA damage.

### Quantitative real-time PCR

Total RNA was extracted from the liver and lung with Trizol reagent (Invitrogen). The cDNA was obtained with reverse transcription PCR. The quantitative real-time PCR was performed with the SYBR Green PCR Master Mix on the Bio-Rad Q5. Primers for ND1, β2M (nDNA), Short fragment (D-loop, mtDNA), and Long fragment (mtDNA) were designed and synthesized by Sangon Biotech (Shanghai, China), as shown in Table S3. GAPDH was used as the internal reference. Relative quantitation of target genes was calculated using the 2^–△△Ct^ method.

### Western blotting

PMVECs were lysed with the RIPA buffer containing protease inhibitor cocktail. After centrifugation and quantification using the BCA protein assay kit, the samples were separated on SDS-polyacrylamide gels and then transferred to PVDF membranes (Millipore). The membranes were blocked for 1 h and subsequently incubated with primary antibodies at 4 °C overnight. Following washing and incubation with secondary antibodies, the bands were visualized using a chemiluminescence gel imaging system.

### Seahorse assay

The oxygen consumption rate (OCR) and extracellular acidification rate (ECAR) measurements were conducted on the Seahorse XFe96 analyzer (Agilent, 102,416-100) as previously described.^33^ Four groups were implemented, namely, sham, CBDL (5%), CBDL plus Agrimoniin (100 μM), and CBDL plus Agrimoniin plus PGC-1α siRNA. PMVECs (1.5 × 10^4^ cells per well) were seeded into each well of 96-well assay plates. After different treatments for 24 h, cells were treated with Seahorse XF medium (Agilent, 103,575-100). The medium was supplemented with 1 mM pyruvate, 2 mM glutamine, and 10 mM glucose for the Mito stress test. The glycolysis stress test included only 2 mM glutamine. In the Mito stress test, cells were treated with 1.5 μM oligomycin, followed by 1 μM FCCP after 15 min, and then 0.5 μM rotenone and antimycin A for 15 min according to the protocol of the Cell Mito Stress Test Kit (Agilent, 103,015-100). The glycolysis stress test involved treating cells with 10 mM glucose, 1.5 μM oligomycin after 15 min, and 50 mM 2-deoxy-d-glucose according to the protocol of the Glycolysis Stress Test Kit (Agilent, 103,020-100). ATP production was calculated from OCR changes upon oligomycin treatment, normalized to baseline respiration. Glycolysis was assessed by ECAR changes, normalized to baseline ECAR. Total protein content was used for normalization.

### Statistical analysis

All measurements are expressed as mean ± standard deviation. Data were analyzed using Student's *t*-test or ANOVA with Bonferroni correction for multiple comparisons between groups (GraphPad Prism 8.0 software, Inc., La Jolla, California, USA). *P*-value <0.05 was considered statistically significant.

## Results

### Imbalanced oxidative phosphorylation/glycolysis correlated with the progression and poor outcome of CLD

Based on the progress of CLD (GSE139602), namely healthy volunteers (*n* = 6), early-stage cirrhosis (*n* = 5), compensated cirrhosis (*n* = 8), decompensated cirrhosis (*n* = 12), and acute-on-chronic liver failure (ACLF, *n* = 8), a trend analysis for gene expression was conducted. Five distinct patterns of gene expression were identified (Fig. 1A). Notably, genes in cluster 3 showed a consistent increase with disease severity, while those in cluster 4 decreased. The largest differential expression was observed between ACLF and early-stage cirrhosis for clusters 1, 2, and 5, whereas clusters 3 and 4 exhibited the most significant differences between ACLF and healthy volunteers. Herein, we selected the interested pathways through GO-BP gene sets and divided them into five categories: angiogenesis, oxidative stress, glucose metabolism, oxidative phosphorylation (OXPHOS), and mitochondria, following the work by Graupera et al.^9^ The heat maps of the interested pathways were plotted in the five clusters (Fig. S1). The top 10 variations between ACLF and healthy volunteers revealed an up-regulation of the angiogenesis and glycolysis pathways during the progression of liver disease (Fig. 1B and C), whereas OXPHOS-related pathways exhibited a down-regulation (Fig. 1D and E). In the current study, we analyzed the in-hospital survival, non-survival, respiratory failure (RF), and non-RF patients with CLD from the MIMIC-IV database. As shown in Table S4, there were 2929 survivors and 580 non-survivors in the CLD patients. Non-survivors had higher CCI, longer length of ICU stay, and higher levels of Lac and LDH (*p* < 0.001). Moreover, higher levels of Lac and LDH during hospitalization were associated with higher in-hospital mortality risk (Fig. 1F). Among the CLD cohort, 1913 patients underwent arterial blood gas analysis, and 1673 met the diagnostic criteria for in-hospital RF (Table S5). Similarly, higher levels of Lac and LDH were associated with an increase in in-hospital RF (Fig. 1G). It is widely accepted that increased levels of Lac and LDH correlate with increased levels of glycolysis. Thus, our data showed that higher levels of glycolysis correlated with poorer outcomes of patients with CLD, particularly pulmonary complications.

As shown in Figure 2A–C, we performed unsupervised clustering based on serum metabolite levels and confirmed that the clusters were consistent with the original groups. Important markers included decreased glucose and increased pyruvic acid levels in the CBDL group (Fig. 2D). Meanwhile, the glycerophospholipid and fructose/mannose metabolism pathways decreased, and ascorbate/aldarate and pyrimidine metabolism pathways increased both by GSVA and GRSA (Fig. 2E–G). Absolute metabolite concentrations showed significant glucose reduction and significant increases in pyruvic acid and pyruvic acid/lactate ratio in CBDL rats (Fig. 2H). Furthermore, pyruvic acid and lactic acid levels increase in advanced liver disease and are indicative of severe extrahepatic complications, such as respiratory failure (Fig. S2). Altogether, imbalanced oxidative phosphorylation/glycolysis correlated with the progression and poor outcome of CLD, suggesting that regulation of oxidative phosphorylation and glycolysis may be a promising integrated therapeutic strategy for mitigating liver and lung injury in HPS.

### Agrimoniin ameliorates liver and lung injury in CBDL rats

To investigate the potential integrated therapy for liver and lung in HPS through modulation of pathological glycolysis, we evaluated the impact of agrimoniin administration on HPS rats. After 2 weeks of CBDL, rats were administered intraperitoneally with agrimoniin daily for 3 weeks (Fig. 3A). Compared with the control group, the CBDL group showed liver pathology like cholestasis and sclerosis, and lung changes with hemorrhagic spots. Agrimoniin treatment alleviated fibrosis and swelling/bleeding in both tissues, normalized liver and lung structure (Fig. 3B), and reduced hyperemia, edema, and tissue proliferation (Fig. 3C). Moreover, agrimoniin alleviated the liver and lung injury, evidenced by the improvement of PaO_2_, P_(A-a)_O_2_, liver enzyme spectrum (Fig. 3D and E). The aforementioned findings suggest that agrimoniin exhibits protective effects on both liver and lung function in HPS.

### Agrimoniin reduces hepatic and pulmonary angiogenesis and the expression of associated proteins in CBDL rats

**Figure 4:**
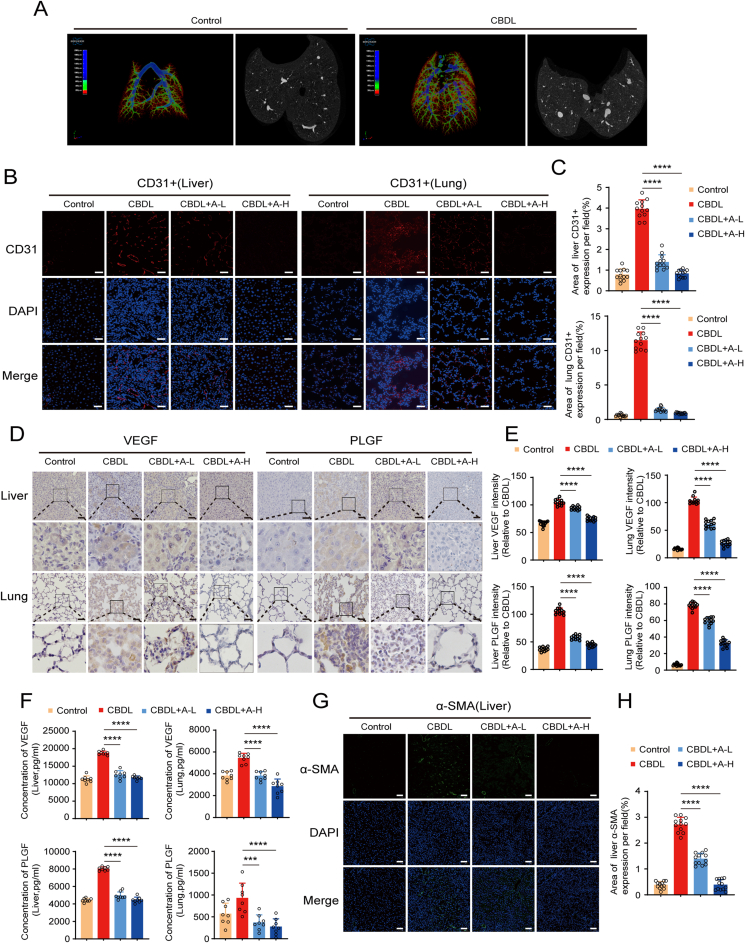
The use of agrimoniin can alleviate hepatic and pulmonary angiogenesis and the fibrosis of liver tissue. **(A)** Vascular casting of lungs and CT images in different groups. **(B, C)** Representative immunofluorescence images of liver and lung sections and comparison of the relative area of CD31 (red) in different groups and its graphical representation (*n* = 12 per group). **(D, E)** Representative VEGF and PLGF immunohistochemistry staining of liver and lung tissue from different groups and their graphical representation (*n* = 12 per group). **(F)** Concentration of growth factors (VEGF and PLGF) of liver and lung tissue in different groups (*n* = 8 per group). **(G, H)** Representative immunofluorescent staining of α-SMA in liver tissue from different groups and its graphical representation (*n* = 12 per group). Nuclei, DAPI (blue). Scale bar, 50 μm ∗*p* < 0.05, ∗∗*p* < 0.01, ∗∗∗*p* < 0.001, and ∗∗∗∗*p* < 0.0001.

As shown in Figure 4A, obvious angiogenesis was observed in the lung of CBDL rats, evidenced by casting microangiography. The CD31 expression in the liver and lungs was also elevated in the CBDL group compared with the control group, while agrimoniin treatment significantly decreased the expression of CD31 (Fig. 4B and C). Immunohistochemistry and ELISA results demonstrated that agrimoniin attenuated the expression of VEGF and PLGF in liver and lung tissues, indicating its potential to inhibit VEGF and PLGF proteins and subsequently reduce angiogenesis (Fig. 4D–F). Compared with the CBDL group, agrimoniin also mitigated the expression of α-SMA in the liver tissue (Fig. 4G and H).

### Agrimoniin presents a protective effect in CBDL rats via regulating glycolysis

**Figure 5:**
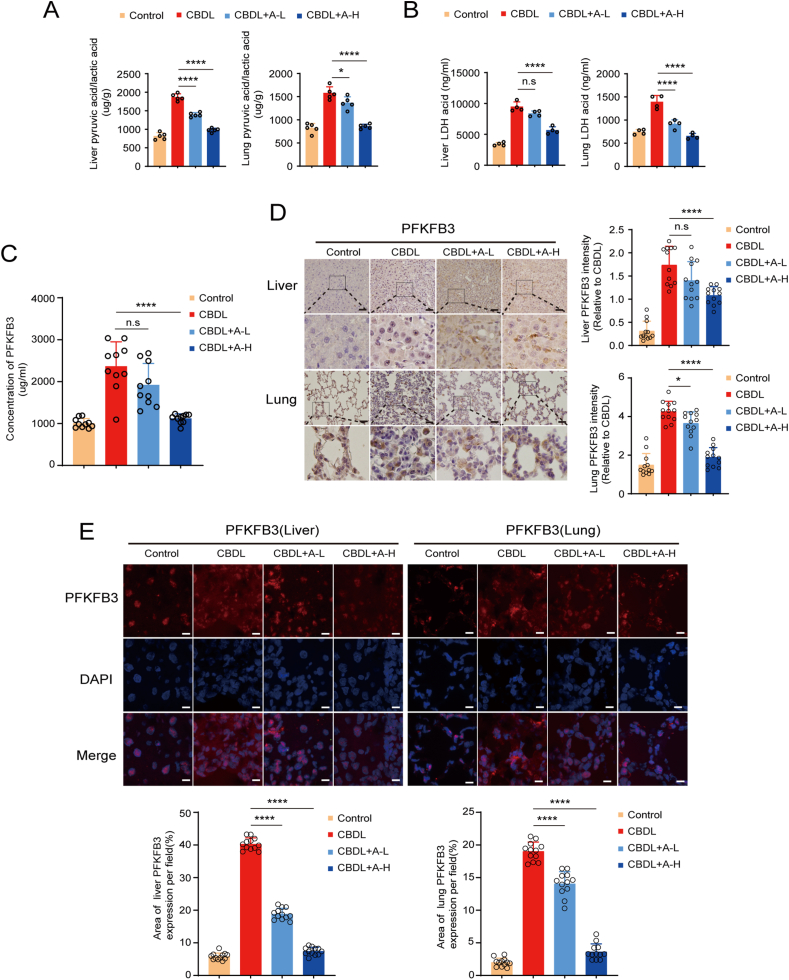
The effect of agrimoniin on glycolytic flux and energy metabolism in CBDL rats. **(A)** The ratio of pyruvic acid/lactic acid in liver and lung tissue (*n* = 5 per group). **(B)** The content of LDH in liver and lung tissue was measured by ELISA (*n* = 4 per group). **(C)** The serum expression of the glycolytic marker protein PFKFB3 in different groups (*n* = 10 per group). **(D)** Representative PFKFB3 immunohistochemistry staining of liver and lung tissue from different groups and its graphical representation (*n* = 12 per group). **(E)** Representative immunofluorescent staining of PFKFB3 in liver and lung tissue from different groups and its graphical representation (*n* = 12 per group). Nuclei, DAPI (blue). Scale bar, 50 μm ∗*p* < 0.05, ∗∗*p* < 0.01, and ∗∗∗∗*p* < 0.0001.

Furthermore, we assessed the ratio of pyruvate and lactic acid in liver and lung tissues in different groups to quantify the glycolytic flux. Agrimoniin treatment significantly reduced the elevated ratio in CBDL rats, with the A-H group exhibiting a more pronounced reduction (Fig. 5A). In comparison to the control group, there was a significant increase in LDH expression in liver and lung tissues, which could be attenuated following treatment with agrimoniin (Fig. 5B). Additionally, the up-regulated glycolytic marker protein PFKFB3 was also reduced by agrimoniin treatment (Fig. 5C and D). In CBDL rats, a significant increase in extra-nuclear PFKFB3 expression was observed. Agrimoniin treatment not only reduced the overall expression of PFKFB3 but also effectively alleviated the ectopic expression of PFKFB3 (Fig. 5E).

### Agrimoniin down-regulates glycolysis via promoting mitochondrial homeostasis

**Figure 6:**
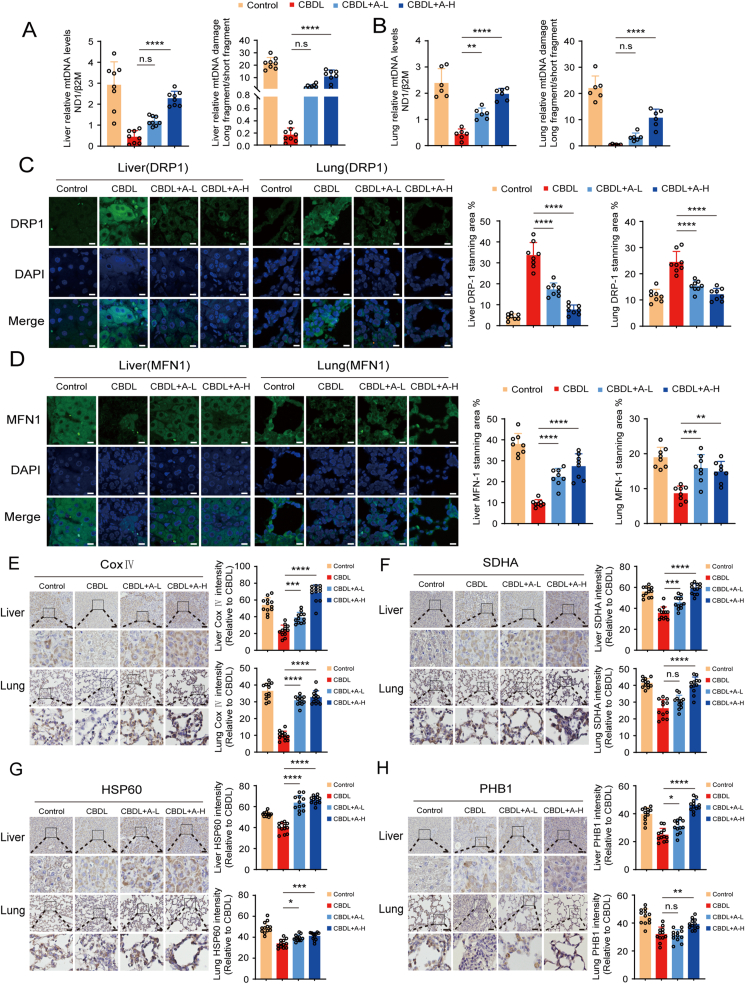
The effect of agrimoniin treatment on mitochondrial homeostasis. **(A)** Quantitative PCR analysis of mtDNA release and mtDNA damage of liver tissue in different groups (*n* = 8 per group). **(B)** Quantitative PCR analysis of mtDNA release and mtDNA damage of lung tissue in different groups (*n* = 6 per group). **(C)** Representative DRP1 immunofluorescent staining of liver and lung tissue from different groups and its graphical representation (*n* = 8 per group). **(D)** Representative MFN1 immunofluorescent staining of liver and lung tissue from different groups and its graphical representation (*n* = 8 per group). **(E)** Representative Cox IV immunohistochemistry staining of liver and lung tissue from different groups and its graphical representation (*n* = 12 per group). **(F)** Representative SDHA immunohistochemistry staining of liver and lung tissue from different groups and its graphical representation (*n* = 12 per group). **(G)** Representative HSP60 immunohistochemistry staining of liver and lung tissue from different groups and its graphical representation (*n* = 12 per group). **(H)** Representative PHB1 immunohistochemistry staining of liver and lung tissue from different groups and its graphical representation (*n* = 12 per group). Scale bar, 50 μm ∗*p* < 0.05, ∗∗*p* < 0.01, ∗∗∗*p* < 0.001, and ∗∗∗∗*p* < 0.0001.

To elucidate the mechanisms underlying the treatment effect of agrimoniin, mitochondrial homeostasis, focusing on mtDNA levels and dynamics of fusion/fission of mitochondria, was analyzed in different experimental groups. The number of long fragments compared with short mtDNA fragments was reduced in CBDL rats, demonstrating that CBDL rats have higher levels of mtDNA damage compared with control rats. Agrimoniin treatment significantly reduced mtDNA damage (Fig. 6A and B).

Subsequently, the mitochondrial fission of dynamin-related protein 1 (DRP1) was observed to be increased in the CBDL group. Conversely, the agrimoniin group exhibited a decrease in DRP1 expression compared with the CBDL group (Fig. 6C). Additionally, an obvious reduction in mitochondrial fusion (mitofusin 1, MFN1) was found in the CBDL group, whereas agrimoniin treatment enhanced MFN1 expression. Notably, the A-H group exhibited greater effects on MFN1 expression compared with the A-L group (Fig. 6D). The transmembrane potential generated by mitochondrial electron transport chains promoted mitochondrial biogenesis. The immunohistochemistry results showed that agrimoniin could restore CBDL-induced decreases in prohibitin 1 (PHB1), succinate dehydrogenase complex flavoprotein subunit A (SDHA), cytochrome c oxidase subunit IV (Cox IV), and heat shock protein 60 (HSP60) to normal levels (Fig. 6E–H).

### PGC-1α is essential for agrimoniin's protective effects on HPS

**Figure 7:**
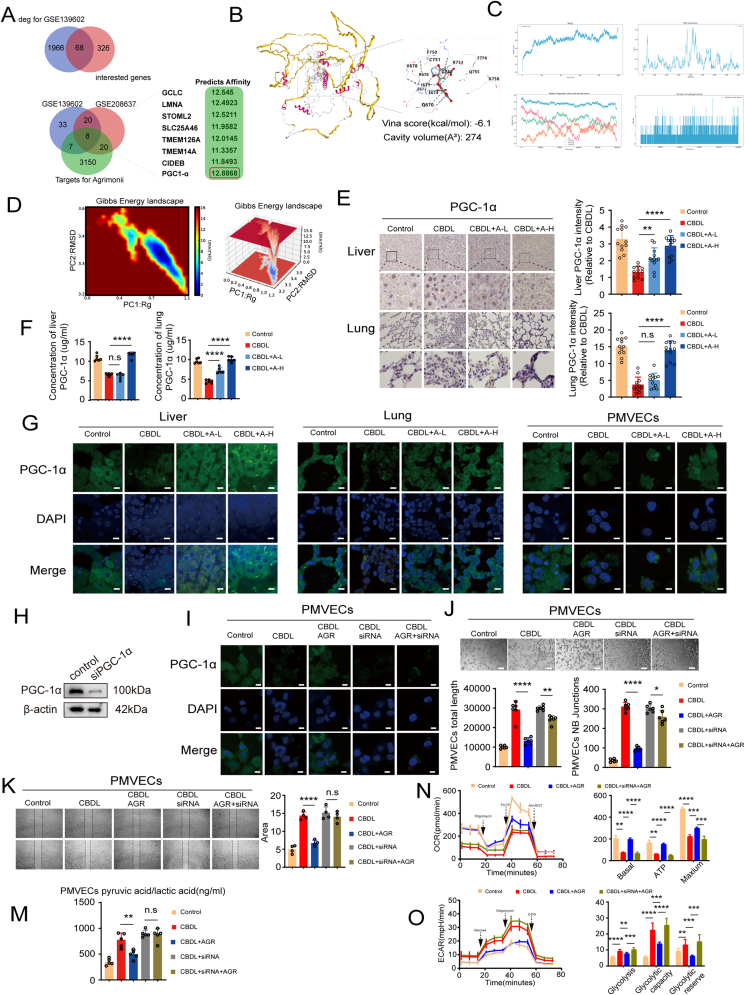
PGC-1α is required for the effects of agrimoniin on hepatopulmonary syndrome. **(A)** Flowchart for predicting potential targets that may be involved in the effect of agrimoniin treatment on hepatopulmonary syndrome. Digital gene expression (DEGs) defined from the pair-wise comparisons were required to satisfy a criterion: the corresponding adjusted *p-*value < 0.05. **(B)** AlphaFold prediction of the 3D structure of the binding between PGC-1α and agrimoniin. **(C)** RMSD of the agrimoniin-PGC-1α complex over 100 ns simulation (top-left); RMSF of residues in PGC-1α (top-right); Radius of gyration (Rg) of the agrimoniin-PGC-1α complex (bottom-left); Hydrogen bond analysis of the agrimoniin-PGC-1α complex (bottom-right). **(D)** The free energy landscape (FEL) assessment of the complex. **(E)** Representative PGC-1α immunohistochemistry staining of liver and lung tissue from different groups and its graphical representation (*n* = 12 per group). **(F)** The ELISA results of PGC-1α in liver and lung tissue (*n* = 6 per group). **(G)** Representative immunofluorescent staining of PGC-1α in liver, lung tissue, and pulmonary microvascular endothelial cells (PMVECs). **(H)** Protein level of PGC-1α in the indicated group. **(I)** Representative immunofluorescent staining of PGC-1α in PMVECs in the indicated groups. **(J)***In vitro* tubule formation assay of PMVECs in the indicated groups and its graphical representation (*n* = 6 per group). **(K)** Migration of PMVECs assessed by scratch wound healing assay in the indicated groups and its graphical representation (*n* = 4 per group). Scratch test scale bar, 500 μm ∗*p* < 0.05, ∗∗*p* < 0.01, and ∗∗∗∗*p* < 0.0001. **(M)** The ratio of pyruvate/lactate in PMVECs by ELISA in indicated groups (*n* = 5 per group). **(N)** The OCR profiles of PMVECs in the indicated groups and quantification of basal respiration, ATP, and maximal respiration. **(O)** Glycolytic profiles of ECAR in the indicated groups and quantification of glycolysis, glycolytic capacity, and glycolytic reserve. Immunofluorescent and immunohistochemistry staining scale bar, 50 μm; tubule experiment scale bar, 200 μm; Scratch test scale bar, 500 μm ∗*p* < 0.05, ∗∗*p* < 0.01, ∗∗∗*p* < 0.001, and ∗∗∗∗*p* < 0.0001.

To identify the potential targets of agrimoniin for HPS, we analyzed differences in glycolysis and oxidative phosphorylation pathways between the two groups with the most significant differences within each cluster, and mapped these to the GO_BP gene set. Patients were divided into three groups: healthy volunteers (*n* = 6), CLD patients (early-stage cirrhosis, compensated cirrhosis, and decompensated cirrhosis, *n* = 25), and the ACLF group (*n* = 8) (GSE139602). By intersecting these differentially expressed genes with the genes of interest, we identified a total of 68 genes (Table S6). In parallel, utilizing the TCM suite, we predicted that agrimoniin could potentially target 3185 genes. Upon further validation of these 68 genes and the potential targets of agrimoniin in the GSE208637 dataset, we ultimately selected 8 genes of significance (Fig. 7A). Moreover, PGC1-α exhibited the highest predicted binding affinity score using CSatDTA predicts affinity (Table S7 and Fig. 7A). Furthermore, the CB-DOCK2 tool was utilized to predict the binding energy between PGC-1α and agrimoniin (Fig. 7B). Subsequent molecular dynamics simulations revealed stable complex formation. As shown in Figure 7C and D, RMSD stabilization at 0.9 Å after 15 ns, RMSF at 0.4 Å, Rg at 3.4 nm with fluctuations < 0.1 nm, and an anisotropy index < 0.25, indicating structural stability and compactness. Over 80% of simulations maintained 1–2 stable hydrogen bonds between the protein and ligand. FEL analysis suggested effective binding of agrimoniin to PGC1-α.

Additionally, the expression of PGC-1α in the CBDL group was significantly reduced, whereas agrimoniin treatment promoted the expression of PGC-1α in liver and lung tissues (Fig. 7E and F). The expression of PGC-1α is predominantly localized in the cytoplasm. Compared with the control group, a significant reduction in PGC-1α expression was observed. Notably, agrimoniin effectively reversed the down-regulation of PGC-1α expression and led to an increase in cytoplasm (Fig. 7G).

We further performed siRNA transfection on PMVECs to inhibit PGC-1α function (Fig. 7H). Accordingly, the activity of agrimoniin treatment, which promotes PGC-1α expression in CBDL rats, was effectively inhibited (Fig. 7I). Tubule formation experiment revealed that agrimoniin treatment significantly reduced total blood vessel length and node formation in the CBDL group. However, this effect was decreased partially by PGC-1α silencing (Fig. 7J). The migration experiment demonstrated similar results that agrimoniin treatment narrowed the PMVEC migration area at 24 h in the CBDL rats, but inhibition of PGC-1α activation increased the migration area compared with the CBDL plus AGR group (Fig. 7K). The requirement of PGC-1α for the protective effect of agrimoniin was further validated by determining the glycolysis flux in PMVECs. PGC-1α inhibition also abrogated the beneficial effect of agrimoniin, as evidenced by ELISA assays of the pyruvate/lactate ratio on PMVECs (Fig. 7M). As shown in Figure 7N, O, the CBDL group exhibited increased ECAR, glycolysis, glycolytic capacity, and glycolytic reverse compared with those in the sham group. Conversely, OCR, OCR basal, OCR ATP, and OCR maximum were reduced in the CBDL group. Agrimoniin treatment effectively reversed these changes induced by HPS serum, but these effects were counteracted by PGC1-α silencing.

Taken together, our data suggest that agrimoniin is a potential integrated therapy for liver and lung in HPS via activation of PGC-1α to inhibit glycolysis and angiogenesis.

## Discussion

**Figure 8:**
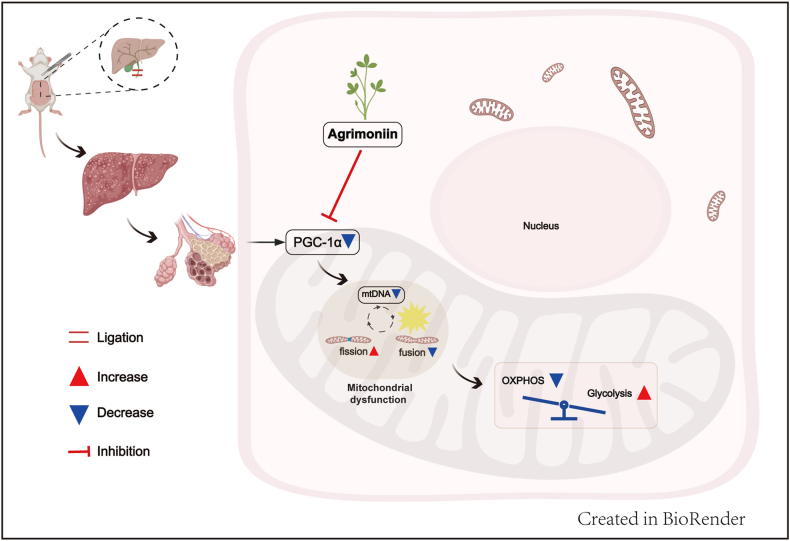
The schematic diagram illustrating the mechanism by which agrimoniin treatment significantly improves liver and lung function by inhibiting pathological angiogenesis and glycolysis via promoting PGC-1α expression to facilitate mitochondrial homeostasis.

There were two main findings in the current study. First, we elucidated the role of impaired glucose metabolism and mitochondrial dysfunction in pathological pulmonary angiogenesis associated with HPS. Second, we demonstrated the therapeutic effects of agrimoniin on HPS by regulating glycolysis and mitochondrial homeostasis via PGC-1α (see Figure 8 for a visual summary).

Pulmonary pathological angiogenesis is a key pathogenic characteristic that contributes to the development of HPS. Inhibiting angiogenesis has been established as an efficacious approach to ameliorate hypoxemia.^34^ Experimental models treated with anti-angiogenic agents, such as sorafenib, have demonstrated improvements in gas exchange and shunting.^35^^,^^36^ Moreover, the anti-angiogenic effects of quercetin significantly improved hypoxemia in CBDL rats.^37^ However, current therapeutic interventions that solely target lung pathology are limited in clinical applications due to significant side effects and poor efficacy in ameliorating hypoxemia. Notably, most hypoxemic symptoms in HPS patients are substantially relieved within one year following liver transplantation. Therefore, treating the liver and lungs together may bring better effects. Our results showed that agrimoniin significantly reduced VEGF, PLGF, and CD31 levels in both lung and liver tissues compared with the CBDL group, validating its anti-angiogenic effects and laying a foundation for exploring its mechanism in HPS. It has been well-documented that pathological angiogenesis interacts with glucose metabolism.^38^ Normally, ATP production from mitochondrial OXPHOS and extramitochondrial glycolysis is approximately 90% and 10%, respectively. However, pathological angiogenesis in vascular endothelial cells relies heavily on aerobic glycolysis, accounting for over 60% of ATP consumption.^10^ Mitochondrial injury and dysfunction, which result in decreased OXPHOS energy supply, lead to the emergence of the extra-mitochondrial glycolytic pathway as the primary source of ATP in cells and tissues, ultimately promoting pathological angiogenesis.^5^ While the liver's central role in glucose homeostasis is well-documented, the impact of disrupted glucose homeostasis on the progression of liver disease and its associated complications has only recently been recognized.^5^^,^^6^ Most functions of the liver are to store large amounts of glycogen and break it down according to the body's needs. Glycogen is a readily available energy reservoir for diverse metabolic processes in the liver.^39^ Its content in the liver is a sensitive indicator of not only cellular energy reserve status but also carbohydrate metabolism in the body.^40^ In the present study, hepatic glycogen disorder was evidenced by the accumulation of glycogen in the liver of CBDL rats, accompanied by drastic weight loss; the OCR and ECAR tests also revealed increased glycolysis and mitochondrial dysfunction. Moreover, PFKFB3 activity promotes glycolysis, with its knockdown reducing glycolytic flux by 40%.^41^ PFKFB3 silencing also diminishes angiogenesis, highlighting glycolysis's crucial role in vascular endothelial cells. Typically, nuclear-localized in normal cells, PFKFB3's overexpression boosts total cellular glycolysis by over 35% and stimulates cell proliferation.^42^ Similarly, our data show that PFKFB3 is increased in CBDL rats, whereas PFKFB3 is alleviated in the presence of agrimoniin. Meanwhile, agrimoniin treatment also ameliorated the disrupted energy metabolism as demonstrated by the OCR and ECAR tests. Thus, we hold the opinion that agrimoniin may ameliorate hypoxemia in HPS by reducing glycolytic flux.

Increasing evidence indicates that mitochondrial damage causes effects of decreased OXPHOS and converts glycolysis into a major extramitochondrial form of energy metabolism to meet the energetic demands of vessel sprouting. Namely, mitochondrial dysfunction promotes glycolytic flux.^43^ The mitochondrial quality homeostasis, which includes both biogenesis, fusion of mitochondria, and fission of damaged mitochondria, as well as mitophagy, is thought to be essential for maintaining a functional energy metabolism. In the present study, we observed that agrimoniin decreases mtDNA damage, regulates the dynamics of fusion/fission of mitochondria, and enhances the expression of mitochondrial electron transport chain proteins. Therefore, agrimoniin reduces glycolytic flux by enhancing mitochondrial quality and homeostasis, thereby constituting an effective treatment strategy for HPS.

Extensive research has established an association between PGC-1α and many metabolic diseases.^44^^,^^45^ Some studies have unveiled its significant role in regulating mitochondrial function in diverse tissues.^46^ In the liver, activated PGC-1α regulates transcription for mitochondrial biogenesis and oxidative phosphorylation, subsequently leading to hepatic gluconeogenesis.^47^ In fact, the transcriptional coactivator PGC-1α has been especially in focus as an important regulator of mitochondrial biogenesis.^48^ Untereiner et al found that hydrogen sulfide induces hepatic mitochondrial biogenesis via PGC-1α signaling pathways, and knockdown of PGC-1α significantly decreased mitochondrial biogenesis in hepatocytes.^49^ In recent years, PGC-1α has been reported to be involved in several signaling pathways modulating mitochondrial biogenesis, such as the AMPK/PGC-1α pathway.^50^ However, PGC-1α not only regulates mitochondrial biogenesis but also exerts extensive effects on the fission and fusion of mitochondria.^51^ The fusion processes that expand mitochondrial networks and fission processes that fragment them play crucial roles in mitochondrial dynamics. The alterations in mitochondrial fission and fusion systems can induce a shift in mitochondrial bioenergetic function from oxidative to glycolytic metabolism.^52^ It is suggested that several mitochondrial fission and fusion proteins, including DRP1 and MFN1, contribute to the regulation of mitochondrial networks. DRP1 assembles into an oligomeric complex on the mitochondrial outer membrane and exerts its role in constricting and dividing mitochondrial membrane segments. The mitochondrial fusion protein MFN1 is primarily localized to the outer mitochondrial membrane and plays a crucial role in the fusion of separate bilayer membranes and the inner mitochondrial membrane. Furthermore, a significant link between PGC-1α and MFN1/DRP1 has been reported by previous studies. In comparison to wild-type mice, PGC-1α-KO mice demonstrate a significant reduction in the expression of the mitochondrial fusion protein MFN1, along with an elevation in the levels of the mitochondrial fission protein DRP1.^53^ Therefore, it is believed that PGC-1α regulates both fusion and fission proteins, influencing the mitochondrial quality homeostasis. In our study, PGC-1α was identified as a potential target of agrimoniin. CBDL disrupted mitochondrial homeostasis, causing mtDNA damage, increased DRP1, decreased MFN1, and reduced PGC-1α expression. These effects were reversed by agrimoniin both *in vivo* and *in vitro*, likely through enhancing PGC-1α to inhibit glycolysis. However, PGC-1α silencing partially attenuated agrimoniin's effects in tubule formation assays, mainly impacting total length and NB junctions. This suggests that PGC-1α, as a transcriptional co-activator, may rely on interactions with other cofactors for mitochondrial regulation, and compensatory mechanisms might be activated when PGC-1α activity is lacking.

This study has some limitations that should be noted. First, the intervention time of agrimoniin was 14 days after the successful production of the CBDL model, and specimens were taken for observation after three weeks of continuous use. Although this is a common observation time for intervention effectiveness in CBDL rats, further observational studies are needed to evaluate the long-term effects of agrimoniin in CBDL rats. Second, agrimoniin is administered intraperitoneally in our experiments. It should be noted that this is not a commonly used method of agrimoniin administration in clinical settings, and further studies are needed to determine the optimal method of administration to achieve maximum efficacy. Last, agrimoniin has not been used as a monotherapy in clinical practice. It is often used in combination with other decoctions clinically. The effect of other drugs on the activity of agrimoniin needs further investigation.

In conclusion, agrimoniin treatment significantly improved liver and lung function by inhibiting pathological angiogenesis and glycolysis via promoting PGC-1α expression to facilitate mitochondrial homeostasis. Agrimoniin emerges as a promising therapeutic candidate for HPS by concurrently targeting hepatic and pulmonary pathologies.

## CRediT authorship contribution statement

**Ziyang Zeng:** Investigation, Funding acquisition, Conceptualization. **Zhiyong Yang:** Writing – review & editing, Writing – original draft, Methodology, Investigation. **Yuhao Lei:** Investigation, Formal analysis, Data curation. **Meiyu Zhou:** Methodology, Investigation. **Lin Chen:** Validation, Project administration, Funding acquisition. **Yang Chen:** Validation, Resources, Funding acquisition. **Xianfeng Wu:** Software, Resources, Formal analysis. **Huiling Cao:** Methodology, Investigation, Formal analysis. **Chunyong Yang:** Validation, Supervision, Conceptualization. **Xiaobo Wang:** Validation, Supervision, Conceptualization. **Karine Belguise:** Validation, Supervision, Conceptualization. **Yujie Li:** Writing – review & editing, Validation, Supervision, Funding acquisition, Conceptualization. **Bin Yi:** Writing – review & editing, Supervision, Funding acquisition, Conceptualization.

## Declaration of generative AI and AI-assisted technologies in the writing process

During the preparation of this work, the author(s) used [ERNIE Bot/POLISH] to improve language and readability. After using this tool/service, the author(s) reviewed and edited the content as needed and take(s) full responsibility for the content of the publication.

## Funding

The work was supported by the 10.13039/501100001809National Natural Science Foundation of China (No. U25A2021 to Bin Yi, No. 82100658 to Yu-jie Li and 82270656 to Lin Chen), the Science Foundation of Chongqing, China (No. cstc2019jcyj-msxmX0667 to Ziyang Zeng), the Youth Top Talent Project of 10.13039/100016834Chongqing Municipal Health Commission (China) (No. YXQN202434 to Yu-jie Li), the Key project of Chongqing Natural Science Foundation (China) (No. CSTB2023NSCQ-ZDX0003 to Bin Yi), and 10.13039/100017501Science-Health Joint Medical Scientific Research Project of Chongqing, China (No. 2025MSXM012 to Yang Chen).

## Conflict of interests

The authors declared no competitive financial interests.

## References

[1] Fallon M.B., Krowka M.J., Brown R.S. (2008). Impact of hepatopulmonary syndrome on quality of life and survival in liver transplant candidates. Gastroenterology.

[2] Del Valle K., DuBrock H.M. (2021). Hepatopulmonary syndrome and portopulmonary hypertension: pulmonary vascular complications of liver disease. Compr Physiol.

[3] Rodríguez-Roisin R. (2023). Hepatopulmonary syndrome: a forgotten liver-induced lung vascular disorder. Arch Bronconeumol.

[4] Rodríguez-Roisin R., Krowka M.J. (2008). Hepatopulmonary syndrome: a liver-induced lung vascular disorder. N Engl J Med.

[5] Moreau R., Clària J., Aguilar F. (2020). Blood metabolomics uncovers inflammation-associated mitochondrial dysfunction as a potential mechanism underlying ACLF. J Hepatol.

[6] Li Z.W., Tu S., Yu X. (2024). Hepatic and extrahepatic metabolic modulation in HBV-related decompensated cirrhosis and acute-on-chronic liver failure. Virulence.

[7] Zhang Y., Tian X.L., Li J.Q., Wu D.S., Li Q., Chen B. (2024). Mitochondrial dysfunction affects hepatic immune and metabolic remodeling in patients with hepatitis B virus-related acute-on-chronic liver failure. World J Gastroenterol.

[8] Mansouri A., Gattolliat C.H., Asselah T. (2018). Mitochondrial dysfunction and signaling in chronic liver diseases. Gastroenterology.

[9] Graupera I., Isus L., Coll M. (2022). Molecular characterization of chronic liver disease dynamics: from liver fibrosis to acute-on-chronic liver failure. JHEP Rep.

[10] Feng J., Li J., Wu L. (2020). Emerging roles and the regulation of aerobic glycolysis in hepatocellular carcinoma. J Exp Clin Cancer Res.

[11] Grochowski D.M., Skalicka-Woźniak K., Orhan I.E. (2017). A comprehensive review of agrimoniin. Ann N Y Acad Sci.

[12] Kashchenko N.I., Chirikova N.K., Olennikov D.N. (2017). Agrimoniin, an active ellagitannin from *Comarum palustre* herb with anti-α-glucosidase and antidiabetic potential in streptozotocin-induced diabetic rats. Molecules.

[13] Fedotcheva T.A., Sheichenko O.P., Fedotcheva N.I. (2021). New properties and mitochondrial targets of polyphenol agrimoniin as a natural anticancer and preventive agent. Pharmaceutics.

[14] Tong Z.H., Guo W.J., Xu Y.J., Zhang Y., Wang W.F. (2025). *Agrimonia pilosa* Extract suppresses NSCLC growth through regulating PI3K/AKT/Bcl-2 pathway. J Ethnopharmacol.

[15] Liu Y., Liu X., Wang H., Ding P., Wang C. (2022). Agrimonolide inhibits cancer progression and induces ferroptosis and apoptosis by targeting SCD1 in ovarian cancer cells. Phytomedicine.

[16] Jang H.H., Bae J.H., Kim M.J., Park M.Y., Kim H.R., Lee Y.M. (2020). *Agrimonia pilosa* ledeb. ameliorates hyperglycemia and hepatic steatosis in ovariectomized rats fed a high-fat diet. Nutrients.

[17] Cho Y.M., Kwon J.E., Lee M. (2018). *Agrimonia eupatoria* L. (agrimony) extract alters liver health in subjects with elevated alanine transaminase levels: a controlled, randomized, and double-blind trial. J Med Food.

[18] Johnson A.E.W., Bulgarelli L., Shen L. (2023). MIMIC-IV, a freely accessible electronic health record dataset. Sci Data.

[19] Subirana I., Sanz H., Vila J. (2014). Building bivariate tables: the compareGroups Package for *R*. J Stat Softw.

[20] Liu Y., Wang W., Zhu P. (2023). Increased non-MAIT CD161^+^CD8^+^ T cells display pathogenic potential in chronic HBV infection. Cell Mol Gastroenterol Hepatol.

[21] Ritchie M.E., Phipson B., Wu D. (2015). Limma powers differential expression analyses for RNA-sequencing and microarray studies. Nucleic Acids Res.

[22] Futschik M.E., Carlisle B. (2005). Noise-robust soft clustering of gene expression time-course data. J Bioinf Comput Biol.

[23] Yang P., Lang J., Li H. (2022). TCM-Suite: a comprehensive and holistic platform for Traditional Chinese Medicine component identification and network pharmacology analysis. Imeta.

[24] Ghimire A., Tayara H., Xuan Z., Chong K.T. (2022). CSatDTA: prediction of drug-target binding affinity using convolution model with self-attention. Int J Mol Sci.

[25] Liu Y., Yang X., Gan J., Chen S., Xiao Z.X., Cao Y. (2022). CB-Dock2: improved protein-ligand blind docking by integrating cavity detection, docking and homologous template fitting. Nucleic Acids Res.

[26] Yang C., Sun M., Yang Y. (2024). Elevated circulating BMP9 aggravates pulmonary angiogenesis in hepatopulmonary syndrome rats through ALK1-Endoglin-Smad1/5/9 signalling. Eur J Clin Invest.

[27] Wilkerson M.D., Hayes D.N. (2010). ConsensusClusterPlus: a class discovery tool with confidence assessments and item tracking. Bioinformatics.

[28] Hänzelmann S., Castelo R., Guinney J. (2013). GSVA: gene set variation analysis for microarray and RNA-seq data. BMC Bioinformatics.

[29] Peng C., Chen Q., Tan S., Shen X., Jiang C. (2024). Generalized reporter score-based enrichment analysis for omics data. Brief Bioinform.

[30] Conway J.R., Lex A., Gehlenborg N. (2017). UpSetR: an R package for the visualization of intersecting sets and their properties. Bioinformatics.

[31] Gu Z. (2022). Complex heatmap visualization. Imeta.

[32] Knutsen R.H., Gober L.M., Sukinik J.R. (2020). Vascular casting of adult and early postnatal mouse lungs for micro-CT imaging. J Vis Exp.

[33] Atas E., Berchtold K., Schlederer M. (2025). The anti-diabetic PPARγ agonist Pioglitazone inhibits cell proliferation and induces metabolic reprogramming in prostate cancer. Mol Cancer.

[34] Miao H., Qiu F., Zhu L. (2021). Novel angiogenesis strategy to ameliorate pulmonary hypertension. J Thorac Cardiovasc Surg.

[35] Yang W., Zhang J., Hu B. (2014). The role of receptor tyrosine kinase activation in cholangiocytes and pulmonary vascular endothelium in experimental hepatopulmonary syndrome. Am J Physiol Gastrointest Liver Physiol.

[36] Kawut S.M., Ellenberg S.S., Krowka M.J. (2019). Sorafenib in hepatopulmonary syndrome: a randomized, double-, placebo-blind controlled trial. Liver Transpl.

[37] Li X., Chen Y., Wang L. (2016). Quercetin alleviates pulmonary angiogenesis in a rat model of hepatopulmonary syndrome. Braz J Med Biol Res.

[38] Gilgenkrantz H., Mallat A., Moreau R., Lotersztajn S. (2021). Targeting cell-intrinsic metabolism for antifibrotic therapy. J Hepatol.

[39] Li L., Zhou H., Wang J. (2023). Metabolic switch from glycogen to lipid in the liver maintains glucose homeostasis in neonatal mice. J Lipid Res.

[40] Bezborodkina N.N., Okovityi S.V., Kudryavtsev B.N. (2021). Postprandial glycogen content is increased in the hepatocytes of human and rat cirrhotic liver. Cells.

[41] Zhu Z., Liu Q., Sun J., Bao Z., Wang W. (2021). Silencing of PFKFB3 protects podocytes against high glucose-induced injury by inducing autophagy. Mol Med Rep.

[42] Bock K.D., Georgiadou M., Schoors S. (2013). Role of PFKFB3-driven glycolysis in vessel sprouting. Cell.

[43] Mei S., Xu Q., Hu Y. (2022). Integrin β3-PKM2 pathway-mediated aerobic glycolysis contributes to mechanical ventilation-induced pulmonary fibrosis. Theranostics.

[44] Qian L., Zhu Y., Deng C. (2024). Peroxisome proliferator-activated receptor gamma coactivator-1 (PGC-1) family in physiological and pathophysiological process and diseases. Signal Transduct Target Ther.

[45] Ramanathan R., Ali A.H., Ibdah J.A. (2022). Mitochondrial dysfunction plays central role in nonalcoholic fatty liver disease. Int J Mol Sci.

[46] Pérez S., Rius-Pérez S., Finamor I. (2019). Obesity causes PGC-1α deficiency in the pancreas leading to marked IL-6 upregulation via NF-κB in acute pancreatitis. J Pathol.

[47] Cheng C.F., Ku H.C., Lin H. (2018). PGC-1α as a pivotal factor in lipid and metabolic regulation. Int J Mol Sci.

[48] Bhargava P., Schnellmann R.G. (2017). Mitochondrial energetics in the kidney. Nat Rev Nephrol.

[49] Untereiner A.A., Fu M., Módis K., Wang R., Ju Y., Wu L. (2016). Stimulatory effect of CSE-generated H_2_S on hepatic mitochondrial biogenesis and the underlying mechanisms. Nitric Oxide.

[50] Popov L.D. (2020). Mitochondrial biogenesis: an update. J Cell Mol Med.

[51] Halling J.F., Pilegaard H. (2020). PGC-1α-mediated regulation of mitochondrial function and physiological implications. Appl Physiol Nutr Metab.

[52] Mishra P., Varuzhanyan G., Pham A.H., Chan D.C. (2015). Mitochondrial dynamics is a distinguishing feature of skeletal muscle fiber types and regulates organellar compartmentalization. Cell Metab.

[53] Halling J.F., Ringholm S., Olesen J., Prats C., Pilegaard H. (2017). Exercise training protects against aging-induced mitochondrial fragmentation in mouse skeletal muscle in a PGC-1α dependent manner. Exp Gerontol.

