# Supplementary material for: Agrimoniin ameliorates intrapulmonary angiogenesis and improves hypoxemia in hepatopulmonary syndrome via PGC-1α activation and glycolysis down-regulation
Source: Genes Dis. 2025 Nov 18;13(5):101941. doi: 10.1016/j.gendis.2025.101941 (PMC13276146; doi:10.1016/j.gendis.2025.101941)
Supplement: Multimedia component 1 [file mmc1.docx]

**Supplementary methods**

**Chemicals and reagents**

HPLC-grade acetonitrile (ACN) and methanol (MeOH) were obtained from Merck (Darmstadt, Germany). MilliQ water (Millipore, Bradford, USA) was used throughout the experiments. Standards were purchased from Sigma-Aldrich (St. Louis, MO, USA) and Zhenzhun. Formic acid was also from Sigma-Aldrich. Stock solutions of standards (1 mg/mL) were prepared in MeOH and stored at -20°C. Before analysis, stock solutions were diluted with MeOH to create working solutions.

**Sample preparation and extraction**

The thawed sample was vortexed for 10 s. Then, 50 μL of the sample was transferred to a centrifuge tube, mixed with 250 μL of 20% acetonitrile/methanol, vortexed for 3 min, and centrifuged at 12,000 r/min for 10 min at 4°C. The supernatant (250 μL) was transferred to a new tube and stored at -20°C for 30 min, then centrifuged again at 12,000 r/min for 10 min at 4°C. Finally, 180 μL of the supernatant was transferred through a Protein Precipitation Plate for LC-MS analysis.

**UPLC Conditions**

The sample extracts were analyzed using an LC-ESI-MS/MS system ((Waters ACQUITY H-ClassD，https://www.waters.com/nextgen/cn/zh.html; MS, QTRAP® 6500+ System, https://sciex.com /). The analytical conditions were as follows.

Amide method: HPLC: column, ACQUITY UPLC BEH Amide (i.d.2.1×100 mm, 1.7 μm); solvent system, water with 10mM Ammonium acetate and 0.3% Ammonium hydroxide (A), 90% acetonitrile/water (V/V)(B); The gradient was started at 95% B (0-1.2 min), decreased to 70% B (8 min),50% B (9-11 min), finaly ramped back to 95% B (11.1-15 min); flow rate, 0.4 mL/min; temperature, 40°C; injection volume: 2 μL.

**ESI-MS/MS Conditions**

A QTRAP 6500+ LC-MS/MS system with an ESI Turbo Ion-Spray interface was used in both positive and negative ion modes, controlled by Analyst 1.6.3 software (Sciex). Parameters: ESI+/-, source temperature 550 ℃, ion spray voltage 5500 V (positive) and -4500 V (negative), curtain gas 35 psi. Metabolites were analyzed by scheduled multiple reaction monitoring (MRM) with optimized declustering potentials (DP) and collision energies (CE). Data acquisition and quantification were performed using Analyst 1.6.3 and Multiquant 3.0.3 software (Sciex).

**Supplementary Figures**


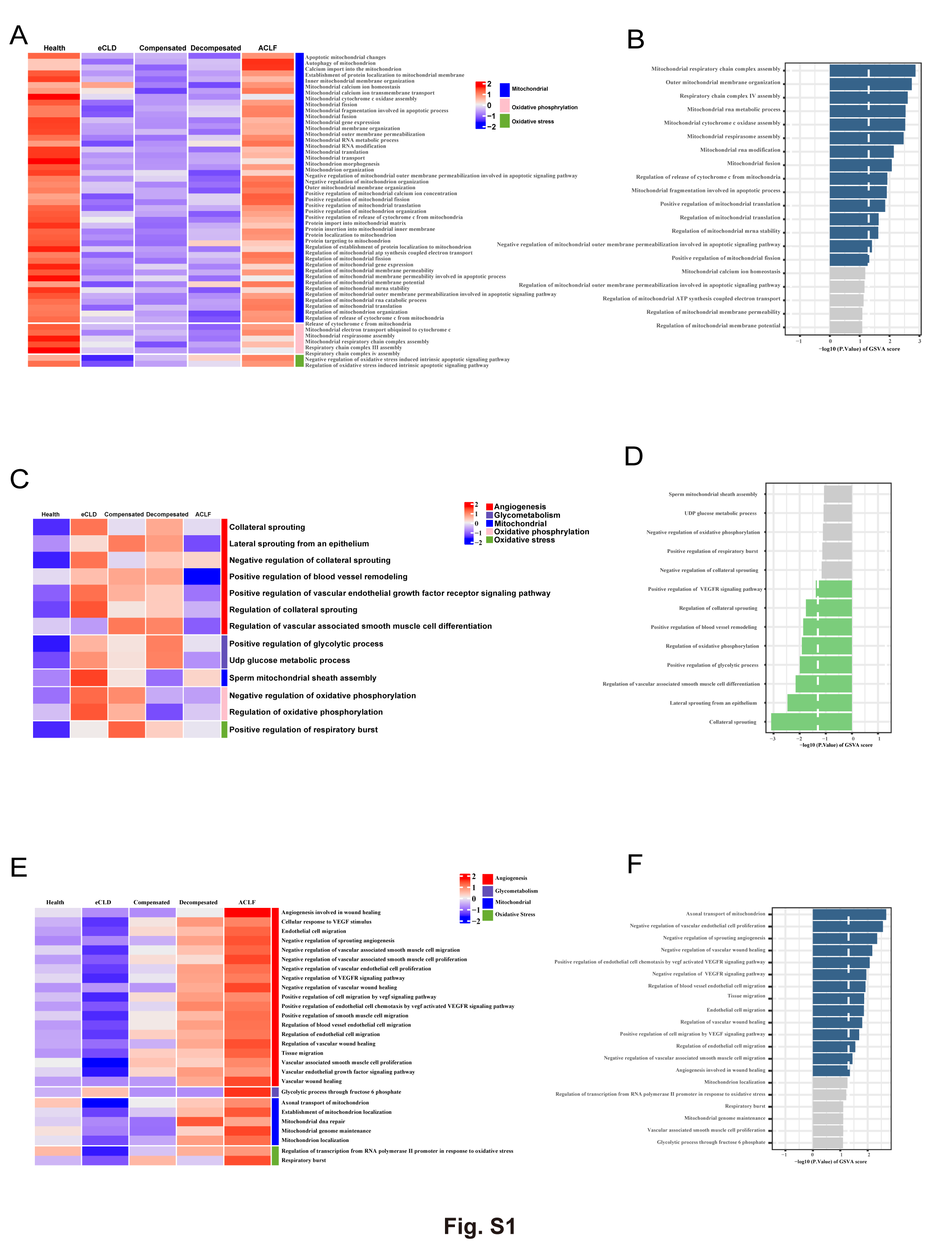


Fig.S1 Based on the progress of CLD (GSE139602).The heat maps of the interested pathways and the top 10 variations were plotted in the cluster1,2,5. (A)Representation of Heat maps for GSVA Cluster 1.(B)The differential pathways of the two most disparate groups in cluster 1.(C)Representation of Heatmaps for GSVA Cluster 2.(D)The differential pathways of the two most disparate groups in cluster 2.(E)Representation of Heatmaps for GSVA Cluster 5.(F)The differential pathways of the two most disparate groups in cluster 5.


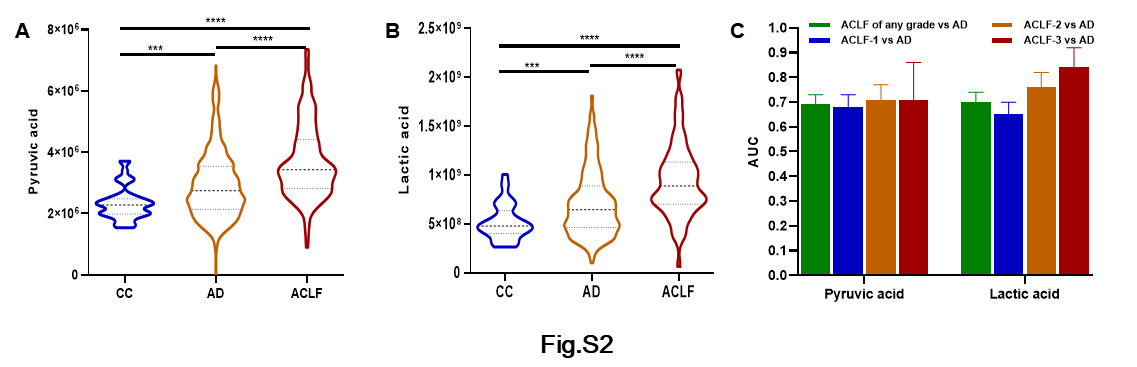


Fig.S2. **The role of circulating pyruvic acid and lactic acid in the process of advanced liver disease.** The data was from the Richard Moreau’s work (*Moreau R, et al. J Hepatol. 2020 Apr;72(4):688-701*). **(A)** The concentration of circulating pyruvic acid in different stage of liver diseases (CC: n=43; AD: n=638; ACLF: n=180). **(B)** The concentration of circulating lactic acid in different stage of liver diseases (CC: n=41; AD: n=638; ACLF: n=173). **(C)** The AUCs of pyruvic acid and lactic acid to distinguish ACLF and AD. CC, compensated cirrhosis; AD, acutely decompensated cirrhosis; ACLF, acute on-chronic liver failure. ACLF-1, Presence of single kidney failure or of any other type of single organ failure, if associated with brain or kidney dysfunction. ACLF-2: Presence of 2 organ failures; ACLF-3, Presence of 3 to 6 organ failures. * means P <0.05, ** means P <0.01, **** means P <0.0001.

**Supplementary Tables**

**Table S1 The tested compounds.**

| **Compounds** | **cpd_ID** | **HMDB** |
| --- | --- | --- |
| L-Aspartate | C00049 | HMDB00191 |
| Fumaric-acid | C00122 | HMDB0000134 |
| cis-Aconitic-acid | C00417 | HMDB00072 |
| Itaconic-acid | C00490 | HMDB0002092 |
| Pyruvic acid | C00022 | HMDB0000243 |
| Lactate | C00186 | HMDB0000190 |
| Alpha-Ketoglutaric Acid | C00026 | HMDB0000208 |
| Succinic Acid | C00042 | HMDB00254 |
| 3-phenyllactic acid | C01479 | HMDB0000779 |
| D-Mannose-6-phosphate | C00636 | HMDB0001078 |
| Dihydroxyacetone-phosphate | C00111 | HMDB0001473 |
| Glycolic-acid | C00160 | HMDB0000115 |
| D(+)-Glucose | C00267 | HMDB0003345 |
| Cysteic-acid | C00506 | HMDB0002757 |
| Glucuronic-acid | C00191 | HMDB0000127 |
| Ureidopropionate | C02642 | HMDB0000026 |
| Gluconate | C00257 | HMDB0000625 |
| Glycerol-3-phosphate | C00093 | HMDB0000126 |
| Glutamine | C00064 | HMDB0000641 |
| L-Asparagine | C00152 | HMDB0000168 |
| L-Alanine | C00041 | HMDB0000161 |
| L-Leucine | C00123 | HMDB0000687 |
| L-citrulline | C00327 | HMDB0000904 |
| 2-Oxoadipic-acid | C00322 | HMDB0000225 |
| Arginine | C00062 | HMDB0000517 |
| Ornithine | C00077 | HMDB0000214 |
| Tyrosine | C00082 | HMDB0000158 |
| Lysine | C00047 | HMDB0000182 |
| Serine | C00065 | HMDB0000187 |
| L-Glutamic-acid | C00025 | HMDB0000148 |
| Threonine | C00188 | HMDB0000167 |
| L-Cystine | C00491 | HMDB0000192 |
| Uracil | C00106 | HMDB0000300 |
| L-2-Hydroxyglutaric-acid-disodium | C03196 | HMDB0000694 |
| Malic-acid | C00149 | HMDB00744 |
| Cyclic-AMP | C00575 | HMDB0000058 |
| Inosine | C00294 | HMDB0000195 |
| Argininosuccinic-acid | C03406 | HMDB0000052 |
| Glyceraldehyde-3-phosphate | C00661 | HMDB0001112 |
| Oxaloacetate | C00036 | HMDB00223 |
| 2-Phospho-D-glyceric acid | C00631 | HMDB0003391 |
| Trehalose-6-phosphate | C00689 | HMDB0001124 |
| Xylulose-5-phosphate | C00231 | HMDB0000868 |
| D-Ribose 5-phosphate-disodium | C00117 | HMDB0001548 |
| D-Fructose-6-phosphate | C00085 | HMDB0000124 |
| D-Ribulose-5-phosphate | C00199 | HMDB0000618 |
| D-ribulose-1,5-bisphosphate | C01182 | HMDB0304322 |
| Succinyl-CoA | C00091 | HMDB0001022 |
| D-Glucose-6-phosphate | C00092 | HMDB0001401 |
| Sedoheptulose-7-phosphate | C05382 | HMDB0001068 |
| D-Glucose-1-phosphate | C00103 | HMDB0001586 |
| 2,3-Diphosphoglyceric acid | C01159 | HMDB0001294 |
| Flavin-mononucleotide | C00061 | HMDB0001520 |
| DL-Glyceric-Acid | C00258 | HMDB0000139 |
| Phosphoenolpyruvic-acid | C00074 | HMDB0000263 |
| 3-phosphoglycerate | C00597 | HMDB0000807 |
| 6-Phosphogluconic-acid | C00345 | HMDB0001316 |
| D-Erythrose 4-phosphate | C00279 | HMDB0001321 |
| Fructose-1,6-bisphosphate | C05378 | HMDB0001058 |
| Acetyl-CoA | C00024 | HMDB0001206 |
| Citric-acid | C00158 | HMDB0000094 |
| IMP | C00130 | HMDB0000175 |
| ADP | C00008 | HMDB0001341 |
| Dihydronicotinamide-adenine-dinucleotide-phosphate(NADPH) | C00005 | HMDB0000221 |
| dTMP | C00364 | HMDB0001227 |
| Adenine | C00147 | HMDB0000034 |
| NicotinaMide-adenine-dinucleotide(NAD) | C00003 | HMDB00902 |
| Triphosphate-guanosine | C00044 | HMDB0001273 |
| AMP | C00020 | HMDB0000045 |
| Guanosine-diphosphate | C00035 | HMDB0001201 |
| UMP | C00105 | HMDB0000288 |
| ATP | C00002 | HMDB0000538 |
| dUMP | C00365 | HMDB0001409 |
| UDP-GlcNAc | C00043 | HMDB0000290 |
| c-di-AMP | C20565 | - |
| Guanosine | C00387 | HMDB0000133 |
| Phosphorylethanolamine | C00346 | HMDB0000224 |
| Isocitric-acid | C00311 | HMDB0000193 |
| dCMP | C00239 | HMDB0001202 |
| dAMP | C00360 | HMDB0000905 |

**Table S2 Information of antibodies**

| **ANTIBODIES** | **SOURCE** | **IDENTIFIER** |
| --- | --- | --- |
| Anti-CD31 | Abcam | Ab119339 |
| Anti-PLGF | Abcam | Ab196666 |
| Anti-VEGF | Proteintech | 19003-1-AP |
| Anti-α-SMA | Abcam | Ab7817 |
| Anti-PFKFB3 | Abcam | Ab181861 |
| Anti-DRP1 | Proteintech | 12957-1-AP |
| Anti-MFN1 | Proteintech | 13798-1-AP |
| Anti-COX IV | Abcam | Ab202554 |
| Anti-SDHA | Abcam | Ab137040 |
| Anti-HSP60 | Abcam | Ab190828 |
| Anti-PHB1 | Proteintech | 10787-1-AP |
| Anti-PGC-1α | Abcam | Ab191838 |

**Table S3 Primer sequences**

| **Gene name** | **Primer sequences(5’-3’)** |
| --- | --- |
| ND1(mtDNA) | Forward:TGGCTCCTTTAACCTCTCCA |
|  | Reverse:GGTTCGGTTGGTCTCTGCTA |
| β2M（nDNA） | Forward:CTGGGTAGCTCTAAACAATGTATTCA |
|  | Reverse:CATGTACTAACAAATGTCTAAAATGGT |
| Short fragment  (D-loop,mtDNA) | Forward:AACCTACCCACCCTTAACAG |
|  | Reverse:CACTCTTGTGCGGGATATTG |
| Long fragment  (mtDNA) | Forward:TCTAAGCCTCCTTATTCGAGCCGA |
|  | Reverse:TTTCATCATGCGGAGATGTTGGATGG |
| Gapdh | Forward:GACATGCCGCCTGGAGAAAC |
|  | Reverse:AGCCCAGGATGCCCTTTAGT |

**Table S4 Comparison of Indicators in Chronic Liver Disease: Survivors vs. Non-survivors**

|  | Survial (n=2929) | In-hospital death (n=580) | *P* |
| --- | --- | --- | --- |
| gender: |  |  | 0.107 |
| F | 1055 (36.0%) | 230 (39.7%) |  |
| M | 1874 (64.0%) | 350 (60.3%) |  |
| Age（y） | 59.1 (13.5) | 63.7 (14.0) | <0.001 |
| Charlson_comorbidity_index | 5.62 (2.93) | 7.06 (2.99) | <0.001 |
| Length of stay in ICU, d | 4.50 (6.37) | 6.62 (8.18) | <0.001 |
| Glu_mean, mg/dL | 132 (35.1) | 142 (45.8) | <0.001 |
| Glu_max, mg/dL | 212 (93.5) | 244 (105) | <0.001 |
| Glu_min, mg/dL | 85.5 (23.9) | 79.0 (35.9) | <0.001 |
| Lac_max, mmol/L | 3.59 (2.67) | 8.55 (5.69) | <0.001 |
| Lac_mean, mmol/L | 2.23 (1.16) | 5.09 (3.95) | <0.001 |
| Lac_min, mmol/L | 1.38 (0.81) | 2.88 (3.21) | <0.001 |
| Ldh_max, IU/L | 731 (1630) | 2136 (4584) | <0.001 |
| Ldh_mean, IU/L | 427 (624) | 1219 (2619) | <0.001 |
| Ldh_min, IU/L | 277 (328) | 738 (1725) | <0.001 |
| ALP_mean, umol/L | 137 (113) | 173 (162) | <0.001 |
| ALT_mean, umol/L | 204 (575) | 386 (816) | <0.001 |
| AST_mean, umol/L | 222 (538) | 803 (1688) | <0.001 |
| Hb_mean，g/L | 9.95 (1.62) | 9.50 (1.56) | <0.001 |
| INR_mean | 1.50 (0.47) | 2.08 (0.76) | <0.001 |
| PT, s | 16.4 (4.84) | 22.5 (8.20) | <0.001 |
| PTT, s | 38.6 (12.6) | 52.3 (18.5) | <0.001 |
| WBC_mean, 10^9^/L | 9.83 (4.89) | 14.1 (11.2) | <0.001 |

**Table** **S5 Comparison of Indicators in Chronic Liver Disease Patients: With vs. Without Respiratory Failure In-Hospital**

|  | **Non-RF (n=240)** | **RF (n=1673)** | **P** |
| --- | --- | --- | --- |
| gender: |  |  | 0.543 |
| F | 88 (36.7%) | 576 (34.4%) |  |
| M | 152 (63.3%) | 1097 (65.6%) |  |
| Length of stay in hospital, d | 14.7 (19.4) | 20.6 (20.7) | <0.001 |
| Age, y | 58.8 (12.3) | 60.4 (13.5) | 0.066 |
| Charlson_comorbidity_index | 5.61 (2.99) | 5.92 (2.73) | 0.140 |
| Length of stay in ICU, d | 3.48 (3.14) | 7.50 (8.73) | <0.001 |
| Glu_mean, mg/dL | 136 (32.1) | 139 (34.3) | 0.323 |
| Glu_max, mg/dL | 223 (84.3) | 245 (98.7) | <0.001 |
| Glu_min, mg/dL | 86.4 (22.2) | 79.4 (25.1) | <0.001 |
| Lac_max, mmol/L | 3.44 (2.85) | 5.51 (4.47) | <0.001 |
| Lac_mean, mmol/L | 2.35 (1.80) | 3.05 (2.59) | <0.001 |
| Lac_min, mmol/L | 1.47 (1.28) | 1.59 (1.79) | 0.191 |
| Ldh_max, IU/L | 685 (1366) | 1346 (3218) | <0.001 |
| Ldh_mean, IU/L | 446 (656) | 726 (1672) | <0.001 |
| Ldh_min, IU/L | 316 (399) | 422 (1034) | 0.012 |
| ALP_mean, umol/L | 121 (85.1) | 143 (124) | 0.004 |
| ALT_mean, umol/L | 229 (598) | 280 (625) | 0.307 |
| AST_mean, umol/L | 245 (516) | 455 (1144) | <0.001 |
| Hb_mean, g/L | 9.91 (1.52) | 9.67 (1.42) | 0.053 |
| INR_mean | 1.53 (0.61) | 1.68 (0.58) | 0.003 |
| PT, s | 16.7 (6.34) | 18.3 (6.22) | 0.002 |
| PTT, s | 38.5 (14.9) | 43.4 (14.8) | <0.001 |
| WBC_mean, 10^9^/L | 10.0 (5.59) | 11.9 (7.56) | <0.001 |
| PaO_2__max, mmHg | 249 (107) | 207 (95.7) | <0.001 |
| PaO_2__mean, mmHg | 185 (63.4) | 117 (35.6) | <0.001 |
| PaO_2__min, mmHg | 144 (67.1) | 72.7 (26.2) | <0.001 |
| Oi_max, mmHg | 414 (55.0) | 341 (104) | <0.001 |
| Oi_min, mmHg | 365 (48.2) | 148 (70.5) | <0.001 |
| Oi_mean, mmHg | 390 (44.2) | 238 (71.2) | <0.001 |

**Table S6 a total of 68 interested genes were selected**

|  | **ACLF.CLD** | **ACLF.health** | **CLD.health** | **AveExpr** | **F** | **P.Value** | **ACLF.CLD** |
| --- | --- | --- | --- | --- | --- | --- | --- |
| **DDIT4** | 1.289827683 | -1.278445571 | -2.568273255 | 7.236681401 | 38.07836031 | 2.58E-10 | 1.45E-07 |
| **PDCD5** | 0.429294134 | -0.697340528 | -1.126634662 | 6.202304354 | 23.17335009 | 1.35E-07 | 1.24E-05 |
| **ETFRF1** | -0.39776742 | -1.560659344 | -1.162891924 | 7.172198048 | 18.43362161 | 1.55E-06 | 7.66E-05 |
| **ATP23** | -0.272143439 | -1.462773149 | -1.190629711 | 4.957405564 | 17.89579046 | 2.08E-06 | 9.57E-05 |
| **SDHAF3** | 0.153558986 | -0.60837053 | -0.761929516 | 5.54102429 | 15.80431576 | 6.78E-06 | 0.000237488 |
| **NDUFAF2** | 0.426966218 | -0.661361816 | -1.088328034 | 6.94403641 | 15.51104156 | 8.04E-06 | 0.000267111 |
| **HIF1A** | 1.111240081 | 1.043425375 | -0.067814706 | 9.87105836 | 15.34611661 | 8.86E-06 | 0.000285536 |
| **DNM1L** | 0.849243551 | 0.837011959 | -0.012231592 | 5.956801223 | 15.22682648 | 9.50E-06 | 0.000300047 |
| **COA1** | 0.320273144 | -0.52941871 | -0.849691853 | 5.537218245 | 15.043238 | 1.06E-05 | 0.000325436 |
| **SLC35F6** | 0.936441145 | 1.068363361 | 0.131922216 | 6.895403498 | 14.72719341 | 1.28E-05 | 0.000373921 |
| **NDUFAF6** | 0.708864636 | -0.045799264 | -0.7546639 | 5.40966491 | 14.57287142 | 1.40E-05 | 0.000401087 |
| **SLC25A46** | 0.12900715 | -0.812397138 | -0.941404287 | 5.314499484 | 14.51997881 | 1.45E-05 | 0.000410623 |
| **SDHAF4** | 0.045292207 | -0.596608489 | -0.641900697 | 5.048904173 | 14.32678741 | 1.63E-05 | 0.000449907 |
| **NDUFS4** | -0.13016604 | -0.794547459 | -0.664381419 | 9.051717223 | 14.24710652 | 1.71E-05 | 0.000463816 |
| **PID1** | 0.034467804 | -1.139417688 | -1.173885492 | 5.016593333 | 13.85763626 | 2.17E-05 | 0.000556047 |
| **MLXIPL** | -0.79006093 | 0.239170177 | 1.029231107 | 9.495419639 | 13.61573097 | 2.51E-05 | 0.000618712 |
| **GHITM** | 0.306482858 | -0.276641306 | -0.583124164 | 4.27506037 | 13.41706799 | 2.84E-05 | 0.000679695 |
| **CHCHD4** | 0.241525974 | -0.48786595 | -0.729391924 | 5.768026632 | 13.3924396 | 2.88E-05 | 0.000687493 |
| **MTX1** | 0.061381968 | -0.868555717 | -0.929937685 | 6.668757572 | 13.21445978 | 3.22E-05 | 0.000741796 |
| **DMAC2** | 0.161064475 | -0.465522836 | -0.62658731 | 4.416707924 | 13.01939569 | 3.64E-05 | 0.000807713 |
| **PLAUR** | 0.904781979 | 0.223137723 | -0.681644255 | 4.489104868 | 12.94981669 | 3.80E-05 | 0.000834382 |
| **IER3** | 2.118683976 | 2.575981646 | 0.45729767 | 6.340770087 | 12.04511329 | 6.77E-05 | 0.001260296 |
| **NDUFB11** | -0.370708725 | -0.97860638 | -0.607897655 | 9.392202346 | 12.01346295 | 6.91E-05 | 0.001281156 |
| **BID** | 0.304122092 | -0.745422015 | -1.049544107 | 6.782742604 | 11.94724804 | 7.21E-05 | 0.00131607 |
| **PRKAA2** | -0.075988815 | 0.968442282 | 1.044431096 | 5.141627815 | 11.38759632 | 0.000103903 | 0.001751094 |
| **HIGD1A** | 0.849143663 | -0.065611664 | -0.914755327 | 6.750903876 | 11.18188687 | 0.00011902 | 0.001955137 |
| **TMEM126A** | 0.344191223 | -0.527791836 | -0.871983059 | 7.846208947 | 11.18111961 | 0.00011908 | 0.001955137 |
| **SLC25A13** | -0.538835928 | -1.070701424 | -0.531865496 | 8.643525042 | 11.08626819 | 0.000126813 | 0.002056504 |
| **RAB5IF** | 0.738085527 | 0.429074098 | -0.309011429 | 6.54177546 | 10.92333381 | 0.000141346 | 0.002231451 |
| **THG1L** | 0.048155631 | -0.541155731 | -0.589311362 | 4.302824813 | 10.90144999 | 0.000143426 | 0.002249619 |
| **HSPA4** | 0.784026357 | 0.648491959 | -0.135534398 | 6.986282456 | 10.6643562 | 0.000168136 | 0.002549788 |
| **PPARGC1A** | -0.086575977 | -1.824936354 | -1.738360376 | 6.242144746 | 10.61456493 | 0.000173869 | 0.00261952 |
| **USP30** | -0.536027715 | -1.195788675 | -0.65976096 | 7.462920028 | 10.38163773 | 0.000203534 | 0.002934255 |
| **COX17** | -0.112923425 | -0.594971201 | -0.482047776 | 10.129379 | 10.30828095 | 0.000213936 | 0.003040435 |
| **PSEN1** | 0.397531252 | 0.793480377 | 0.395949125 | 8.163030156 | 10.24115136 | 0.000223943 | 0.003140814 |
| **LYRM2** | 0.622695741 | 0.473139776 | -0.149555965 | 6.51760822 | 10.01486648 | 0.000261438 | 0.003499907 |
| **NDUFV2** | 0.086242566 | -0.619350613 | -0.705593179 | 10.08602597 | 9.974852775 | 0.000268724 | 0.003562641 |
| **GCLC** | -0.25207159 | -0.940801471 | -0.688729881 | 6.641344377 | 9.768062581 | 0.000309913 | 0.003937002 |
| **UQCC1** | 0.065495118 | -0.443581095 | -0.509076213 | 4.292385727 | 9.59124694 | 0.000350362 | 0.00430575 |
| **TMEM14A** | -0.433069132 | -1.495723439 | -1.062654307 | 8.087046157 | 9.573407049 | 0.000354739 | 0.004330728 |
| **NDUFB2** | 0.189697234 | -0.326040933 | -0.515738167 | 10.34977533 | 9.525089563 | 0.000366884 | 0.004440834 |
| **NDUFA2** | -0.118657689 | -0.496905219 | -0.378247531 | 9.340559494 | 9.467787837 | 0.000381852 | 0.004587917 |
| **RHOA** | 0.521647682 | 0.66847653 | 0.146828848 | 10.04174429 | 9.365114823 | 0.000410291 | 0.004842485 |
| **COX16** | 0.047459209 | -0.836538329 | -0.883997539 | 8.267646176 | 9.350660794 | 0.000414469 | 0.004882437 |
| **SMIM20** | 0.282650464 | -0.351847905 | -0.634498368 | 7.10825603 | 9.335106817 | 0.000419015 | 0.004924809 |
| **FAM162A** | -0.750974852 | -1.288589715 | -0.537614863 | 9.361402268 | 9.183889575 | 0.000466023 | 0.005346532 |
| **TOMM6** | 0.367643455 | -0.18580687 | -0.553450325 | 6.683043891 | 9.119790563 | 0.000487584 | 0.005542258 |
| **NDUFA5** | -0.188820435 | -0.992033208 | -0.803212773 | 6.854418305 | 9.100776224 | 0.000494179 | 0.005596553 |
| **COA5** | -0.131045148 | -0.582920197 | -0.451875049 | 5.430598084 | 9.050821418 | 0.000511952 | 0.005731073 |
| **LMNA** | 0.563412994 | 1.045391698 | 0.481978704 | 7.013274548 | 8.666672176 | 0.000673082 | 0.007005391 |
| **HDAC6** | -0.591577539 | 0.080968133 | 0.672545672 | 8.331866976 | 8.648187762 | 0.000682061 | 0.00706582 |
| **STOML2** | 0.252778073 | -0.438426218 | -0.691204291 | 8.14969247 | 8.611804112 | 0.000700105 | 0.007200243 |
| **TOMM70** | 0.417421206 | -0.13899309 | -0.556414296 | 4.567480324 | 8.585620665 | 0.000713398 | 0.007300374 |
| **ATP5F1E** | 0.292982228 | 0.330149936 | 0.037167707 | 11.20362064 | 8.564809187 | 0.000724152 | 0.007385871 |
| **CHCHD3** | 0.51307417 | -0.028875448 | -0.541949617 | 7.015216012 | 8.492751707 | 0.000762715 | 0.007681629 |
| **NDUFB6** | -0.025531137 | -0.775386566 | -0.749855429 | 8.697370754 | 8.468731544 | 0.000776042 | 0.007769253 |
| **CLU** | -0.083229722 | -0.448349485 | -0.365119763 | 3.992381757 | 8.446248756 | 0.000788737 | 0.00787075 |
| **MCU** | 0.731069103 | 0.494474551 | -0.236594552 | 5.921543414 | 8.443054028 | 0.000790558 | 0.007884666 |
| **UQCRC2** | 0.218986174 | -0.854296459 | -1.073282633 | 7.766696125 | 8.440032394 | 0.000792285 | 0.007897624 |
| **SDHD** | 0.108585085 | -1.484579294 | -1.593164378 | 6.512084403 | 8.436737402 | 0.000794173 | 0.007903198 |
| **SLC25A18** | -0.851725955 | 0.068124599 | 0.919850554 | 8.82466398 | 8.426615067 | 0.000800001 | 0.007932706 |
| **FOXRED1** | -0.130647266 | -0.988808232 | -0.858160966 | 6.122169957 | 8.323444584 | 0.000862016 | 0.00838899 |
| **NDUFB3** | 0.246053412 | -0.442476343 | -0.688529755 | 6.390011006 | 8.273799308 | 0.00089363 | 0.008646539 |
| **FIS1** | 0.03421282 | -0.643986356 | -0.678199176 | 8.856158284 | 8.234595005 | 0.000919451 | 0.008804136 |
| **PPP2CA** | 0.514582124 | 0.015042583 | -0.499539542 | 5.341190744 | 8.216120515 | 0.000931888 | 0.008890961 |
| **BAX** | 0.381131311 | 1.051607756 | 0.670476445 | 5.289914293 | 8.183173843 | 0.000954506 | 0.009061319 |
| **ATP5PB** | 0.061006745 | 0.360604893 | 0.299598147 | 11.27866743 | 8.0970244 | 0.001016403 | 0.009424688 |
| **CIDEB** | -0.616831557 | -1.393984386 | -0.777152829 | 8.236837665 | 7.998511347 | 0.001092359 | 0.009924721 |

**Table S7 CSatDTA Predicts Affinity of Eight Gene-Expressed Proteins to Agrimonia**

| **Gene symbol** | **Uniprot ID** | **Predicted binding affinity** |
| --- | --- | --- |
| GCLC | P48506 | 12.545 |
| LMNA | P02545 | 12.4923 |
| STOML2 | Q9UJZ1 | 12.5211 |
| SLC25A46 | Q96AG3 | 11.9582 |
| TMEM126A | Q9H061 | 12.0145 |
| TMEM14A | Q9Y6G1 | 11.3357 |
| CIDEB | Q9UHD4 | 11.8493 |
| PPARGC1A | Q9UBK2 | 12.8868 |
